# Modelling temperature-dependent properties of polymorphic organic molecular crystals†
†Electronic supplementary information (ESI) available: The Nyman Polymorph Library, additional results, molecular flexibility rules, melting point regression analysis, a description of co-prime splitting and force field parameters for halogens. See DOI: 10.1039/c6cp05447a



**DOI:** 10.1039/c6cp05447a

**Published:** 2016-10-31

**Authors:** Jonas Nyman, Graeme M. Day

**Affiliations:** a School of Chemistry , University of Southampton , Southampton , UK . Email: g.m.day@soton.ac.uk ; Tel: +44 2380599174

## Abstract

We present a large-scale study of the temperature-dependence of structures, free energy differences and properties of polymorphic molecular organic crystals. Lattice-vibrational Gibbs free energy differences between 475 pairs of polymorphs of organic molecular crystals have been calculated at 0 K and at their respective melting points, using a highly accurate anisotropic multipole-based force field and including thermal expansion through the use of a (negative) thermal pressure. Re-ranking of the relative thermodynamic stability of the polymorphs in each pair indicates the possibility of an enantiotropic phase transition between the crystal structures, which occurs in 21% of the studied systems. While vibrational contributions to free energies can have a significant effect on thermodynamic stability, the impact of thermal expansion on polymorph free energy differences is generally very small. We also calculate thermal expansion coefficients for the 864 crystal structures and investigate the temperature-dependence of mechanical properties, and pairwise differences in these properties between polymorphs.

## Abstract 2

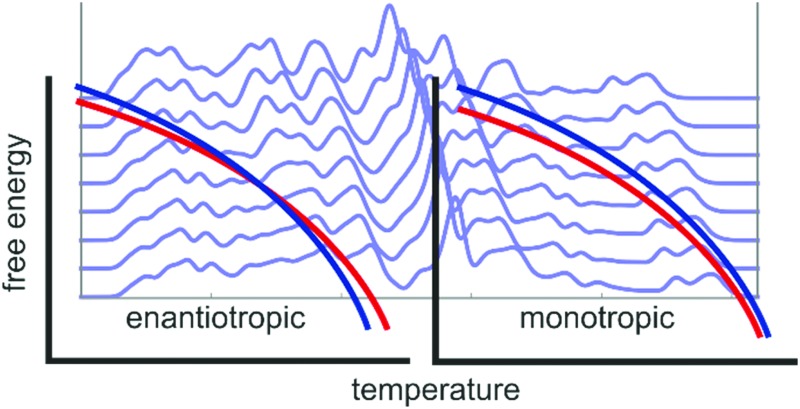
We examine the importance of thermal expansion on relative stabilities and properties of crystalline polymorphs.

## 1 Introduction

Polymorphism, *i.e.* the possibility that a compound can occur in several distinct crystalline phases,^1,2^ has attracted much interest because of its potential implications for the development and production of pharmaceuticals,^3–5^ organic semiconductors,^6^ explosives,^7,8^ chocolates,^9^ and other molecular materials where solid state properties are important. It is now well established that polymorphism is common, occurring in about half of all studied compounds with no strong correlation to the size or flexibility of the molecule.^10^

Polymorphism occurs because different crystalline packing arrangements can result in nearly the same free energy.^1,2,11–14^ Kinetic factors affecting the crystallization of different polymorphs, and the mechanisms by which their phase transitions are facilitated, are less well understood.^15^

At any given pressure, each polymorph pair is either enantiotropic or monotropic. In monotropic pairs, one polymorph has a larger free energy than the other at all temperatures (below the melting point), *i.e.* the higher energy polymorph is always thermodynamically unstable and will eventually transform to the more stable form. Consequently, monotropic transitions are irreversible and exothermic. Once a transition has occurred, it is sometimes nearly impossible to obtain the less stable form again.^16,17^

In enantiotropic pairs, both polymorphs can be thermodynamically stable, but in different temperature intervals. At a particular temperature, both polymorphs have exactly the same free energy, which can manifest as solid-to-solid phase transitions, iso-energetic polymorphism^18^ and concomitant crystallisation.^19^ Enantiotropic transitions are in principle reversible. The transition is endothermic when going from the low-temperature form to the high-temperature polymorph. However, there are often significant deviations from ideal thermodynamic behaviour, caused by lattice defects, impurities and kinetic barriers.^20^ Crystals can be kinetically stable for apparently indefinite time and reversible transitions usually display significant hysteresis.

It is possible to computationally study the relative stability of, and transitions between, polymorphs through molecular dynamics simulations (MD).^21,22^ The simulation can be augmented with metadynamics to allow a global exploration of free energy minima and transition barriers.^23,24^ The lattice vibrations, in the form of a phonon density of states *g*(*ω*) can be obtained from the velocity autocorrelation function over a molecular dynamics trajectory,^25^ and this has the advantage of directly including anharmonic effects, such as thermal expansion. The main difficulties lie in the sampling of long wavelength phonons, requiring large simulation cells, and in performing molecular dynamics with an energy model that is accurate enough. Lattice dynamics offers an alternative to MD simulations that can be much more efficient, but in addition to the problem of sampling low-frequency modes,^26^ difficulties arise in the treatment of anharmonic vibrations and thermal expansion.

To calculate realistic free energy rankings of molecular crystals it is usually necessary to employ periodic dispersion-corrected density functional approximations (DFT-D),^27^ fragment based QM/MM methods^28,29^ or anisotropic force fields based on distributed multipoles.^30–32^

We have recently demonstrated that the difference in lattice vibrational energy between polymorphs is typically very small but can be as large as 4 kJ mol^–1^ at room temperature and that in 9% of cases this is large enough to reverse their relative stability.^14^ This article is to a large extent a follow-up study of our earlier results, focusing on the temperature-dependence of polymorphic relative stabilities.

The small energy differences between polymorphs makes it challenging to correctly rank their relative stabilities, making for instance crystal structure prediction (CSP) difficult.^33^ To meet the challenge, we have improved our methods in several ways. We have benchmarked our force fields to verify their accuracy. The force field used in this study is comparable to periodic PBE with Grimme's D2 or the Tkatchenko–Scheffler dispersion corrections and similar methods.^32^ Our previously described method for Brillouin zone sampling by linear supercells has been supplemented with a Debye model to treat dispersion of acoustic phonons. In addition, we use a kernel density method to model dispersion of optic phonons, which improves the convergence of the calculated thermodynamic properties.^32,34^ In this study we also consider some effects of the anharmonicity in the lattice vibrations; the thermal expansion is modelled in the quasi-harmonic approximation (QHA),^35^ using the thermal pressure method.^36^

Few studies have been made of the anharmonic vibrational contribution to polymorph free energy ranking. The CSP study of glycol and glycerol by van Eijck^37^ is a rare exception. In examining their influence on the ranking of predicted structures, it was found that the harmonic vibrational zero point and thermal vibrational energy can have an important effect, but that the energetic impact of thermal expansion generally does not significantly affect rankings. Because of the computational cost and the small size of the effect it was concluded that it may be preferable to neglect thermal expansion in CSP. More recently, Heit and Beran applied high level fragment-based quantum chemical methods to investigate the importance of thermal expansion in modelling the thermodynamic properties of four small molecule crystal structures.^38^ The errors in free energies that they associated with ignoring thermal expansion were small, due to cancellation of opposing effects in enthalpy and entropy.

The number of molecular crystals for which the importance of thermal expansion has been assessed is currently very small. Therefore, we feel that a larger sample of polymorph pairs is needed to estimate its importance in modelling the energetics and properties of organic molecular crystals. The large sample studied here should provide good estimates of general trends among polymorphs.

The influence of thermal expansion on polymorph relative stabilities will depend on how different the volumetric thermal expansion coefficients are between polymorphs, and this in turn depends on differences in elastic properties of the materials. Since elastic properties are also of interest in the formulation and production of pharmaceutical tablets, we examine the shear and bulk moduli, and their temperature-dependence.

In this study, we present results of rigid-body lattice-vibrational free energy differences at 0 K and at the respective melting point temperature for a large number of organic molecular polymorphic crystals. In doing so, we hope to be able to estimate how common it is that relative stabilities are re-ranked by temperature, *i.e.* we are estimating how common enantiotropism is, and how large lattice energy differences can be overcome by thermal contributions to the free energy.

## 2 Methods

The main objective of this study is to use a large selection of known polymorph pairs and for each pair calculate the difference in lattice-vibrational free energy and other properties at 0 K and at the melting point temperature *T*_m_. We determine how often temperature causes a re-ranking of the thermodynamic stability, and obtain an estimate for how commonly polymorph pairs are enantiotropically related.

Below we describe the set of polymorph pairs and the calculations we have performed on them. We describe how the structures were energy-minimised on a realistic potential energy surface and how rigid-body harmonic (HA) and quasi-harmonic approximation (QHA) lattice dynamics calculations were carried out. Finally, we explain how melting point temperatures were obtained for all the crystal structures.

Although the main focus is on general trends in the large set of polymorph pairs, we also performed more detailed studies of a selected set of crystals. The calculated properties of individual systems are compared to experimental literature data, allowing us to estimate the overall accuracy of our results.

### 2.1 Polymorph selection

We started from a set of 1061 single-component organic molecular packing polymorphs of 508 compounds that we have described previously.^14^ We have used the best experimentally determined structure of each polymorph.^39^ These are polymorphs of compounds containing the elements H, C, N, O, F, S and Cl in any combination up to *ca.* 55 atoms per asymmetric unit. The methods presented here could be applied to molecular salts or solvates, but we choose to focus on single-component systems in this study. The compounds and crystal structures are referred to by their Cambridge Structural Database (CSD) reference codes.^40^ Crystals with a non-integer number of molecules in the asymmetric unit (*Z*′) have had their symmetries lowered to obtain whole molecules in the asymmetric unit.

From this set we had to exclude a number of structures for various reasons; see the ESI.† All studied structures are packing polymorphs where the molecular conformers in each pair do not differ by more than RMSD = 0.25 Å in atomic coordinates, as calculated with Tormat,^41^ ignoring hydrogen positions.

The final structure set is included in the ESI† as CIF files and as a list of CSD reference codes. The set consists of 864 crystal structures of 418 different compounds that successfully completed all calculations. There are 391 polymorph pairs, 26 triplets and 1 quadruplet for a total of 475 pairwise comparisons. This set is ideal for computational studies of polymorphism. The ESI† contains additional information about these systems, such as SMILES strings,^42^ InChI identifiers and the chemical formula of the compounds, calculated thermal pressures, experimental and predicted melting points, elastic moduli, volume thermal expansion coefficients, unit cell volumes, *Z* and *Z*′-values. We offer this data, the Nyman Polymorph Library (NPL2016), in the hope it will facilitate further computational studies of polymorphs.

### 2.2 Energy model

The lattice energy consists of the intermolecular cohesive energy and a quantum chemical evaluation of the intramolecular energy, calculated with dispersion-corrected density functional theory (DFT-D).

1 *E*_latt_ = *E*atom–atominter + *E*DFT-Dintra

The intermolecular cohesive energy between any two molecules M and N is calculated as a sum over atom–atom pair interactions:2
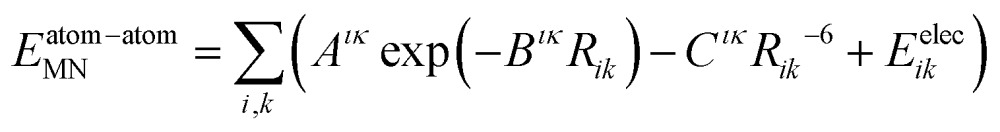
where *i* and *k* are atoms of type *ι* and *κ* belonging to molecules M and N, respectively and separated by the distance *R*_*ik*_. The first two terms model the short range repulsive and attractive non-electrostatic intermolecular interactions, with force field parameters *A* ^*ικ*^, *B* ^*ικ*^ and *C* ^*ικ*^.

We have used the W99rev6311 and W99rev6311P force fields,^43^ supplemented with parameters for sulfur,^44^ fluorine and chlorine. Halogen atoms were modelled with an anisotropic repulsion,^45^ using parameters provided in the ESI.†

The intramolecular electronic structure and energy is calculated with Gaussian09 at the B3LYP/6-311G(d,p) level of theory, with a Becke–Johnson damped version of Grimme's D3 dispersion correction.^46,47^

The final term *E* ^elec^, describing electrostatic interactions, was calculated from atom-centred multipoles up to rank 4 (hexadecapole) on all atoms,^30,31^ obtained from a distributed multipole analysis^48^ of the molecular electron density using Gdma 2.2.06.^49^ The multipoles may be polarized by performing the DFT calculation in a polarizable continuum model (PCM).^50,51^ Charge–charge, charge–dipole and dipole–dipole interactions were calculated with Ewald summation. All other interactions were calculated between whole molecules with a centre of mass distance less than 20 Å.

The type of hybrid force field/DFT energy model used here is highly accurate and may even be competitive with GGA DFT-D methods, such as PBE-D2, in reproducing absolute lattice energies.^32^ Furthermore, calculations of polymorph energy differences benefit from an important cancellation of systematic errors in the force field.

### 2.3 Flexible molecule optimisation

Crystal geometry optimisations were performed with CrystalOptimizer ^52,53^ version 2.4.2. CrystalOptimizer minimises the lattice energy (eqn (1)) while allowing a set of selected intramolecular degrees of freedom (bond lengths, angles and dihedrals) and unit cell dimensions to change in response to intermolecular forces. All other intramolecular degrees of freedom are optimised to their DFT-D equilibrium values. The optimisation is performed numerically by iterating between inter- and intramolecular calculations. Intermolecular packing forces were calculated with Dmacrys 2.0.4, using the W99rev6311 force field and distributed multipoles, as described above.

We used an in-house method to automatically select flexible degrees of freedom from the default *Z*-matrix generated by CrystalOptimizer, as previously described,^14^ although for this study we have improved the selectivity for 3-, 4- and 5-membered rings (see the ESI†).

At the DFT level of theory we are using, crystals with more than 55 atoms per asymmetric unit, or crystals of large flat molecules or macrocycles are often prohibitively expensive to optimize with CrystalOptimizer due to convergence difficulties in calculating the molecular Hessian matrix. These problematic structures were excluded.

### 2.4 Lattice dynamics

We have re-optimised the crystal structures obtained from CrystalOptimizer and performed rigid-body lattice dynamics using the W99rev6311P force field.^43^ This force field was parametrised specifically to be used together with multipoles derived from a charge density obtained in a polarizable continuum model (PCM). A relative permittivity of *ε*_r_ = 5.0 was used in this study.

For all crystal structures, we calculated the lattice-vibrational free energy at 0 K and at the respective melting point for each structure with both the harmonic and quasi-harmonic methods, as described in the ESI† and our previous article.^32^ These calculations make use of a Debye approximation for the acoustic phonon dispersion near the Brillouin zone centre, and a Gaussian kernel density estimate (KDE) of dispersion around all calculated phonon frequencies.

At non-zero temperatures we calculate the free energy in the harmonic approximation, *A* ^HA^(*T*), as the sum of the lattice energy and the vibrational energy, *F*HAvib(*T*), from the phonon frequency density of states calculated at the fully relaxed structure

3 *A*^HA^(*T*) = *E*_latt_ + *F*HAvib(*T*)

The thermal expansion caused by zero-point vibrations is implicitly included in the empirical force field, which was parametrised to fit lattice energy minima to low temperature crystal structures. Therefore, we calculate the harmonic (HA) and quasi-harmonic (QHA) free energy at 0 K as:

4 *A*^QHA^(0) = *A*^HA^(0) = *E*_latt_ + ZPE

The harmonic approximation is based on an assumption that phonon frequencies and the crystal structure do not change with temperature. Our previous results using the harmonic model^14^ have shown that vibrational contributions can alter polymorph relative stabilities by up to about 2 kJ mol^–1^, which is mostly due to entropy differences. These contributions are significant since the static energy difference between pairs of polymorphs is found to be less than 2 kJ mol^–1^ in over half of the molecules investigated. However, at temperatures near the melting point the harmonic model could lead to unacceptable errors. We have previously described our implementation of a quasi-harmonic method to account for thermal expansion and the temperature-dependence of lattice vibrations.^32^

Thermal expansion and free energies in the quasi-harmonic approximation are calculated using the thermal pressure method,^36^ where a thermal pressure is calculated from the change in vibrational energy with volume, *P*_th_(*T*) = –∂*F*_vib_(*T*)/∂*V*. Geometry optimisation in the rigid body approximation at –*P*_th_ results in a thermally expanded crystal structure close to the free energy minimum at constant pressure. The quasi-harmonic free energy *A* ^QHA^ can then be calculated for the thermally expanded structure as

5 *A*^QHA^(*T*) = *E*_latt_(*T*) + *F*QHAvib(*T*)

All lattice dynamics calculations were performed in Dmacrys 2.0.4,^31^ using algorithms that have been described elsewhere.^32,54–56^ Full details of the free energy calculations are provided in the ESI.†

For a number of structures it was not possible to obtain a valid energy minimum. The structures MALOAM01, SIRMIQ and SALIAZ02 drastically changed structure when the thermal pressure was applied and were excluded. In addition, 42 polymorph families were excluded because one or more of the structures became unstable at negative pressures, exhibiting imaginary phonon frequencies and indefinite elastic tensors.

Elastic moduli were calculated for a crystalline aggregate of each structure by averaging over the calculated elastic tensors, for which we used the Hill average.^57^ The thermal expansion is expected to lead to significant softening, lowering the bulk and shear moduli as the temperature is raised. This effect is investigated between 0 K and the melting point. Due to the importance of mechanical properties for processing at room temperature, thermal expansion calculations were repeated for the entire structure set at 298 K, to also determine room temperature bulk and shear moduli distributions.

### 2.5 Melting point predictions

Melting points are known for many of the polymorphs in the set. The melting temperature differences between polymorphs are smaller than the required accuracy for our purposes. Therefore, we have only attempted to determine one melting point per polymorph family. We have used experimentally determined melting temperatures whenever this was reported in the CSD. This was the case for 225 (44%) of the 508 initially selected polymorph families.

For all other polymorph families, we have predicted the melting points using two different methods. The first method is based on molecular fragment contributions, and is implemented in the Mtbtnt program in the EPASuite ^58^ software by the US Environmental Protection Agency. The program internally uses two algorithms for estimating the melting temperature and returns a weighted average of these. The first algorithm is based on Joback's method.^59^ The second algorithm estimates the melting point as *T*_m_ = 0.5839·*T*_b_ as suggested by Gold and Ogle,^60^ where *T*_b_ is the boiling point, which is also calculated from fragment contributions using a method by Stein and Brown.^61^ The Mtbtnt program takes SMILES strings^42^ as input. These were obtained from atomic coordinates *via* the Open Babel software.^62^

However, molecular fragment based methods are notoriously imprecise and the standard deviation of the error in estimated melting points is 63.9 K.^58^ We have therefore attempted to improve the predicted temperatures by using a second method relying on the correlation between lattice enthalpies and melting points. A straight proportionality is sometimes assumed between these quantities,^63^ but we do not find this to be correct. A good fit can be obtained after a variance-stabilising Anscombe transformation in the form of a square root.^64^ A non-linear regression analysis in Minitab 17 between the 225 previously obtained experimental melting points and the crystals' intermolecular energies *E*_inter_ yields the following model for the melting points:6
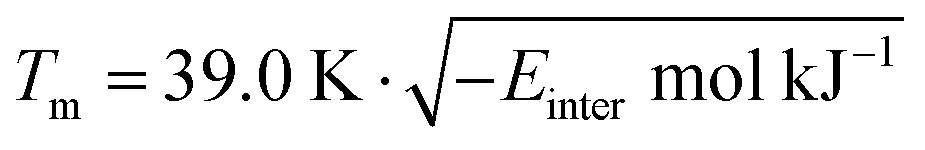
The intermolecular energies were taken from our previous study.^14^ The residual standard deviation for this model is 54.5 K, and we note that this very simple regression model yields more accurate melting point predictions than the molecular fragment method. Details of the regression and residual analysis are included in the ESI.†

We have used the mean of these two methods as our predicted melting temperatures. We estimate the mean absolute error of this average to be 39 K, or less than 10%. Predicted and experimental melting temperatures are included as ESI.†

### 2.6 Detailed study of selected systems

We performed a more in-depth study of a few selected systems. These were chosen primarily as families with several experimentally well characterized polymorphs.

Plenty of experimental data have been reported for polymorphs of the following compounds: acridine (ACRDIN),^65,66^ 1,2-ethanediamine (ETDIAM),^67,68^ *m*-nitrophenol (MNPHOL),^69–71^ acetaminophen/paracetamol (HXACAN),^72,73^ glutaric acid (GLURAC),^74–76^ octa-sulfur (FURHUV),^77–80^ adipic acid (ADIPAC),^81,82^ 2,2′-dipyridylamine (DPYRAM),^83^ theophylline (BAPLOT),^84–89^ and pyrazine-2-carboxamide (PYRZIN).^90^

Polymorphs of these compounds were studied computationally in greater detail. We have calculated free energy curves, thermal expansion rates and phonon densities of states that can be compared to published experimental data.

The lattice energy was calculated relative to the energy of the gas phase molecular conformer. Continuous free energy curves were obtained by hermite piecewise polynomial interpolation,^91^ using both the free energy and analytic first derivatives (entropies) at several temperatures.

## 3 Results and discussion

We first present the results of studies of selected crystal structures. Comparisons to experimental data allows us to estimate the overall accuracy of the calculations. We then show general trends in the whole set of polymorph pairs. We direct the reader to the ESI† for additional figures that could not be included here.

### 3.1 Results for particular systems

For polymorphs of selected compounds we calculated free energy curves, the thermal expansion and phonon densities of states which can be compared to published experimental data. Only a few results are shown here, most results are included in the ESI.†

**Fig. 1:**
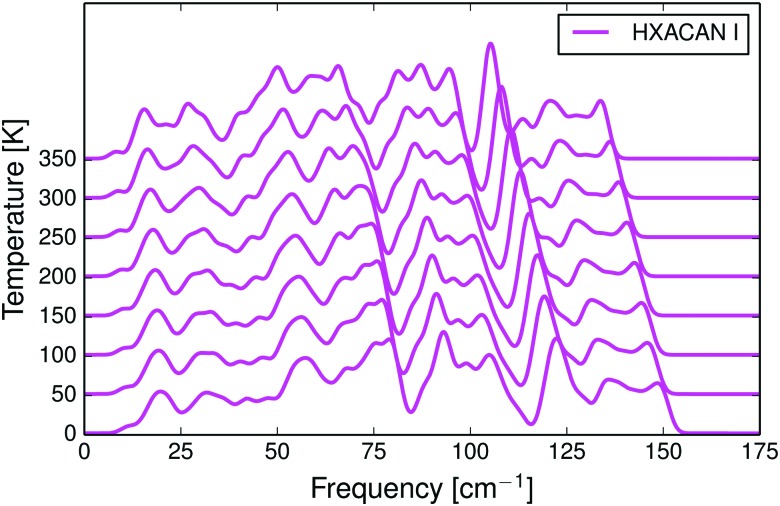
Calculated phonon density of states for the monoclinic phase I of paracetamol as a function of temperature. The thermal expansion causes a shift in the density of states towards lower frequencies.

In the harmonic approximation, the phonon frequencies are assumed not to change with temperature. In reality, the vibrations soften as the molecules move apart due to thermal expansion, resulting in a shift toward lower frequencies. This frequency shift is reproduced well with the quasi-harmonic approximation. In Fig. 1 we show the calculated temperature dependence of the phonon density of states in paracetamol form I (HXACAN12). Corresponding figures for crystals of *m*-nitrophenol, glutaric acid, sulfur and 1,2-ethanediamine are included in the ESI† (Fig. S4–S7). The temperature dependence of phonon frequencies was compared to low-frequency variable-temperature Raman spectra.^68,70,80^ In all cases we see a realistic softening of the vibrational frequencies with increasing temperature, often in quantitative agreement with experiment.

**Fig. 2:**
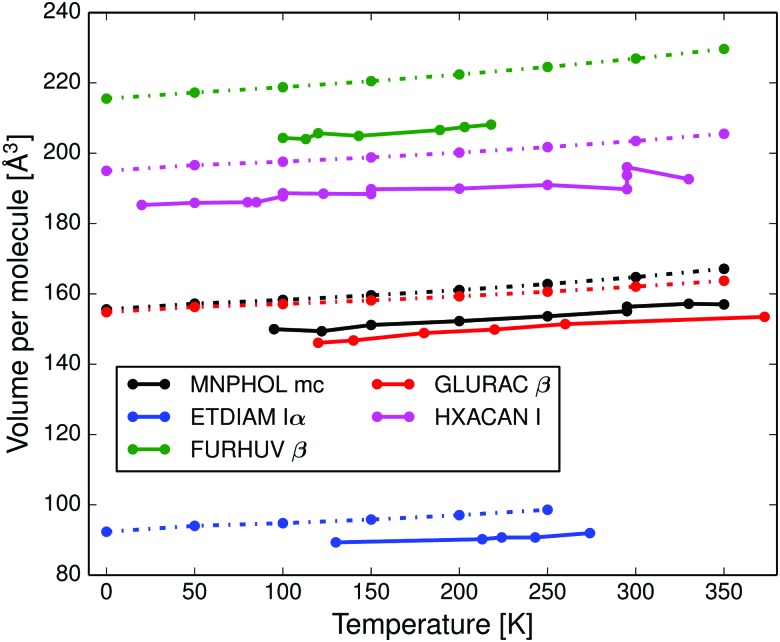
Calculated volumetric thermal expansion (dashed lines) compared to experimental reference data (solid lines) of: monoclinic *m*-nitrophenol (MNPHOL mc); 1,2-ethanediamine form Iα (ETDIAM Iα); β-sulfur (FURHUV β); glutaric acid form β (GLURAC) and paracetamol form I (HXACAN I).

In Fig. 2 and Fig. S8–S16 (ESI†) we compare the calculated anisotropic and volumetric thermal lattice expansion of the same five crystal structures to experimental data. All five structures have too large unit cell volumes, caused by a known systematic error in the force field.^32,43^ The mean absolute relative error in calculated thermal expansion coefficients is estimated to be 15% based on data for 9 crystal structures, see Table 1, and the calculations do not systematically over- or underestimate the thermal expansion (the mean signed error is 2.5%). The coefficients were calculated as a linear thermal expansion between 0 to 350 K for predictions (0 to 250 K for ETDIAM, which melts at 284 K) and by a linear regression of experimental data in the CSD over the indicated temperature ranges.

**Table 1:** Calculated and experimentally observed volumetric thermal expansion coefficients in units of 10^–6^ K^–1^. The temperature range is the range used for the experimentally determined structures used in the regression; CSD reference codes for the structures used are given in the ESI. Errors are quoted for all experimental values from the standard errors of the regression, apart from DPYRAM, for which only two structures are available. Room temperature structures are taken as 293 K

Structure	Calc. α	Exp. α	Temp. range [K]
MNPHOL mc	211.9	214.6 ± 14.9	95–350
ETDIAM Iα	270.5	193.0 ± 38.7	130–274
FURHUV β	187.3	154.6 ± 24.6	100–218
GLURAC β	164.6	206.6 ± 21.5	120–373
HXACAN I	154.4	156.9 ± 15.3	20–330
ADIPAC I	173.8	198.4 ± 11.3	100–293
DPYRAM mc	178.9	164.8	150–293
PYRZIN β	155.3	181.1 ± 12.3	90–293

			MA%E = 15.0%

**Fig. 3:**
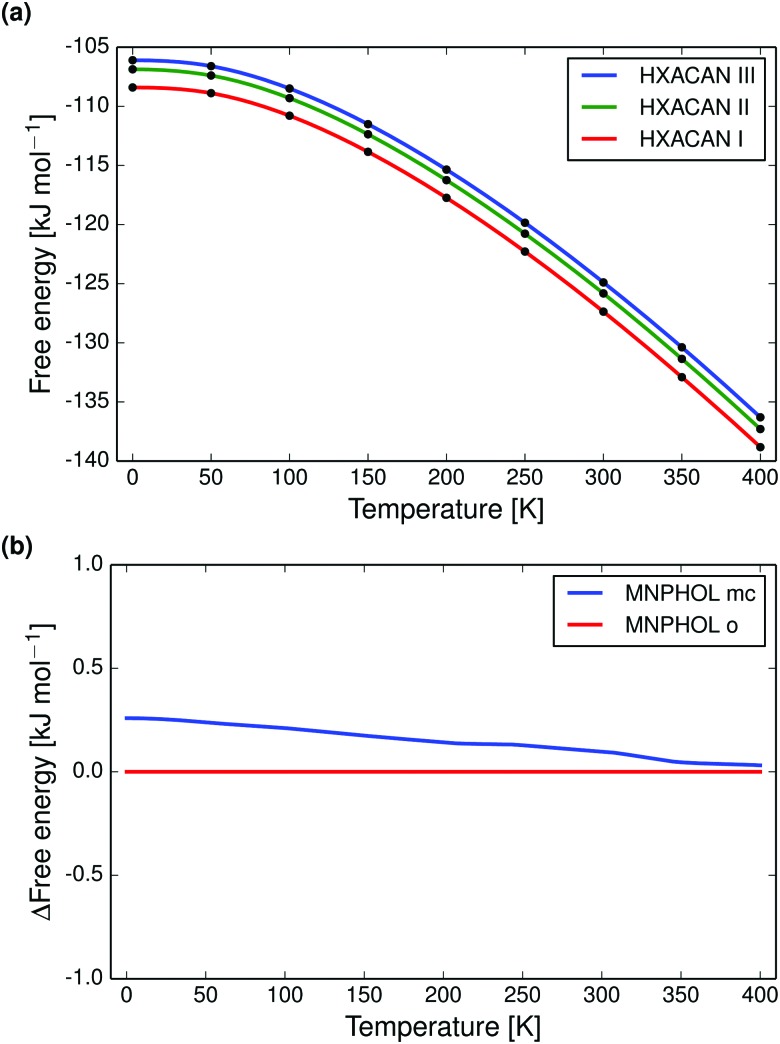
Quasiharmonic calculated free energy curves of polymorphs of paracetamol and *m*-nitrophenol. (a) Shows the calculated absolute free energy *vs.* temperature curves for polymorphs I, II and III of paracetamol, which are correctly found to be monotropic and in the experimentally observed stability order. (b) Shows the calculated difference in free energies between the monoclinic (mc) and orthorhombic (o) forms of *m*-nitrophenol. The known enantiotropic transition at 350 K is almost reproduced.

Full quasi-harmonic free energy curves were drawn for polymorphs of paracetamol (Fig. 3a), *m*-nitrophenol (Fig. 3b), acridine, adipic acid, 2,2′-dipyridylamine, theophylline, and pyrazine-2-carboxamide (Fig. S17–S26, ESI†).

Paracetamol forms I, II and III we find to be monotropically related (at zero pressure, Fig. 3a), in agreement with experimental data.^92,93^ The monoclinic polymorph of *m*-nitrophenol has a larger entropy than the orthorhombic form and the free energy curves almost reproduce the known enantiotropic transition point at 350 K (Fig. 3b).

Calculated vibrational energies and entropies are realistic and sometimes in good agreement with experimental data. The complete free energy curves are however often not in agreement with experimental data. This is primarily because of errors in the 0 K relative lattice energies; if polymorphs are incorrectly ranked by lattice energy the free energy relationship will also be wrong.

### 3.2 General trends among polymorphs

In the end, 864 crystal structures were successfully free-energy minimised. The structures constitute 391 pairs, 26 triplets and 1 quadruplet of polymorphs, for a total of 418 polymorph families and 475 pairwise comparisons. Experimentally known melting points were used for 174 families (42%) and for 244 families we have used predicted melting temperatures. We find no differences in the trends between the sets with experimentally known and predicted melting points.

**Fig. 4:**
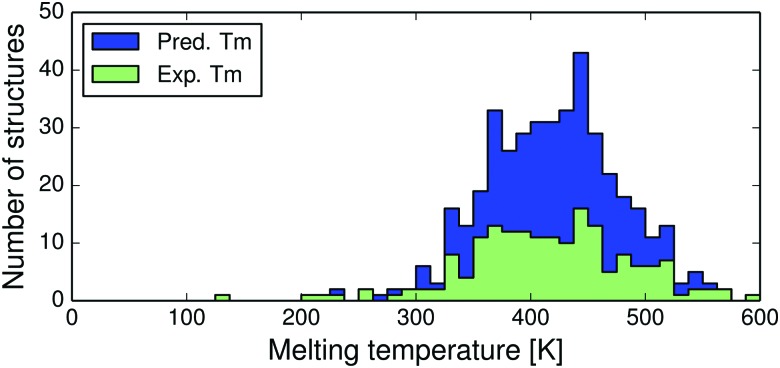
Distributions of experimental (green) and predicted (blue) melting point temperatures for the 418 polymorph families.

Most crystal structures in this study have melting points between 350 and 470 K, see Fig. 4. Since these temperatures are substantially higher than room temperature, we expect to see a larger vibrational contribution to the free energy and more re-ranking of polymorph stabilities compared to previous room temperature studies.^13,14^

#### 3.2.1 Thermal expansion and mechanical properties

**Fig. 5:**
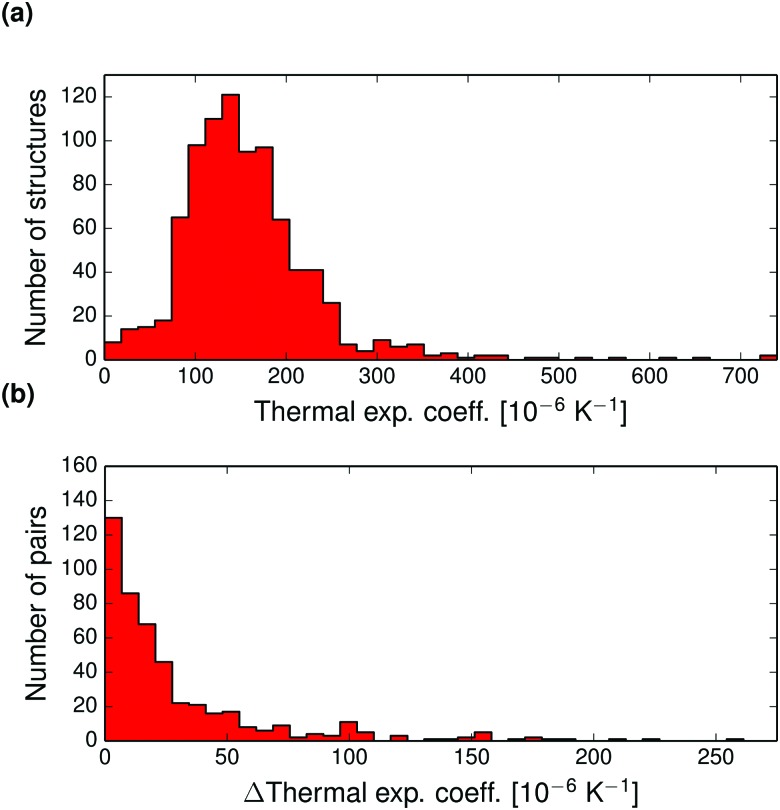
(a) Distribution of calculated volumetric thermal expansion coefficients in the set of 864 crystal structures. (b) Pairwise absolute differences in volumetric thermal expansion coefficients for 475 polymorph pairs.

The volumetric thermal expansion coefficient of each crystal structure was calculated as a finite difference between the 0 K and the thermally expanded structure. Fig. 5a displays the distribution of expansion coefficients for the whole set of structures. The thermal expansion coefficient is typically between 80–240 × 10^–6^ K^–1^, corresponding to an expansion of 0.8 and 2.4% per 100 K, which can be used as a rule of thumb for organic molecular crystals in general. The largest thermal expansions are seen for weakly bound crystals of organic solvents, such as acetonitrile (QQQCIV01 and QQQCIV08, 747 and 749 × 10^–6^ K^–1^), chloroethane (XAXCOQ and XAXCOQ02, 614 and 563 × 10^–6^ K^–1^), cyclobutanol (KETVEK01 and KETVEK03, 408 and 528 × 10^–6^ K^–1^) and 2,5-dimethylpyrazine (WIZFEQ and WIZFEQ01, 648 and 208 × 10^–6^ K^–1^).

The pairwise differences in volumetric thermal expansion coefficient between polymorphs are shown in Fig. 5b. Differences in thermal expansion coefficients are less than 15.8 × 10^–6^ K^–1^ for 50% of pairs, and rarely differ by more than 50 × 10^–6^ K^–1^. So, while thermal expansion tends to be similar between polymorphs, the differences can sometimes be relatively large.

**Fig. 6:**
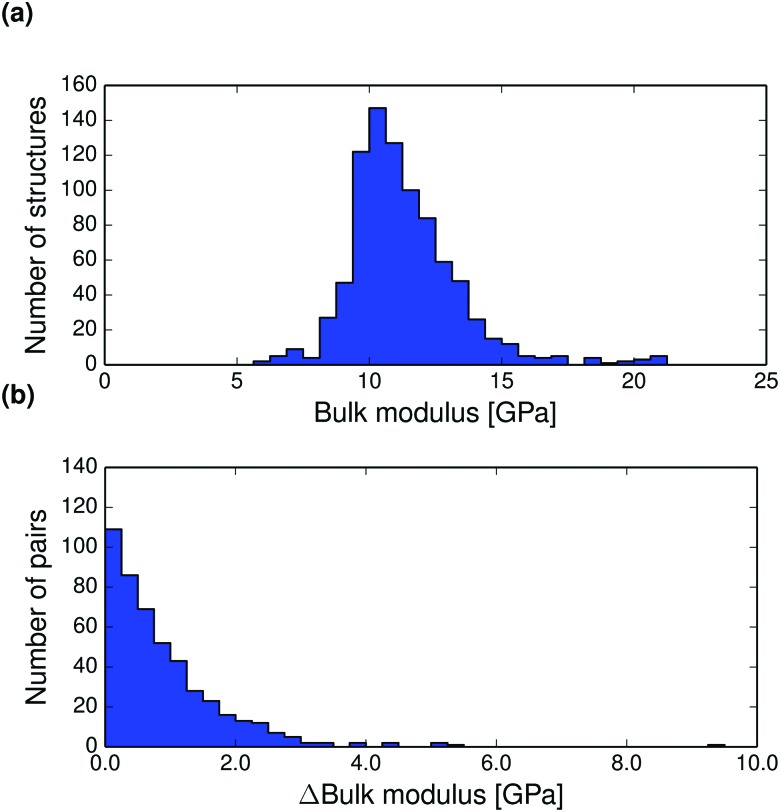
(a) Distribution of *T* = 0 K calculated bulk moduli for the entire set of crystal structures. (b) Pairwise absolute differences in bulk modulus between polymorphs at 0 K.

**Fig. 7:**
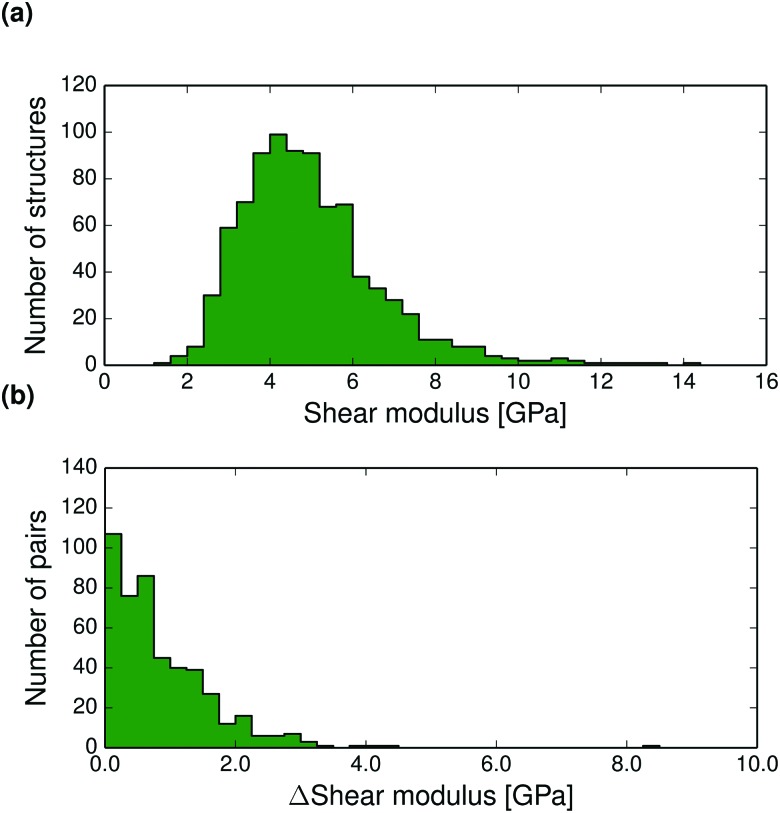
(a) Distribution of *T* = 0 K calculated shear moduli for the entire set of crystal structures. (b) Pairwise absolute differences in shear modulus between polymorphs at 0 K.

The thermal expansion of a crystal is related to its elastic properties and differences in bulk and shear moduli are often of interest, for example in the manufacturing of drug tablets. The Hill averaged bulk and shear moduli calculated for all structures at 0 K are displayed in Fig. 6a and 7a. The calculated bulk and shear moduli for small organic molecule crystalline aggregates are usually 8–15 GPa and 2–8 GPa respectively at low temperature.

Bulk modulus differences between polymorphs exceed 2 GPa in only 10% of polymorph pairs and are less than 0.5 GPa in 41% of pairwise comparisons, see Fig. 6 (and Fig. S27–S28 for corresponding differences calculated at 298 K, ESI†). These small differences in the resistance to volume changes partly explain why polymorphs also tend to have similar thermal expansion coefficients. We find that the shear moduli also rarely differ by more than 2 GPa between polymorphs of the same compound, although this corresponds to a larger relative difference because of the typically smaller values of shear moduli. Since low shear moduli can relate to facile plastic deformation, the relatively large differences in shear moduli (Fig. 7 and Fig. S29 and S30 at 298 K, ESI†) demonstrate why polymorph differences have important consequences for the tabletability of pharmaceuticals.^94^

Pairwise absolute differences in elastic moduli between polymorphs at 0 K are shown in Fig. 6b and 7b.

#### 3.2.2 Free energies

The thermal expansion leads to a softening of vibrational modes and hence an increase in vibrational entropy. This is counteracted by a less favourable lattice energy with increasing volume. The resulting total energetic effect of thermal expansion can be calculated as the difference between the quasi-harmonic free energy *A* ^QHA^ and the harmonic approximation free energy *A* ^HA^. This difference, calculated at the melting point of each structure, is displayed in Fig. 9a. Based on this large set of structures, we calculate that thermal expansion typically contributes only 1.7 kJ mol^–1^ and never more than 4 kJ mol^–1^ to the total free energy of organic molecular crystals.

**Fig. 8:**
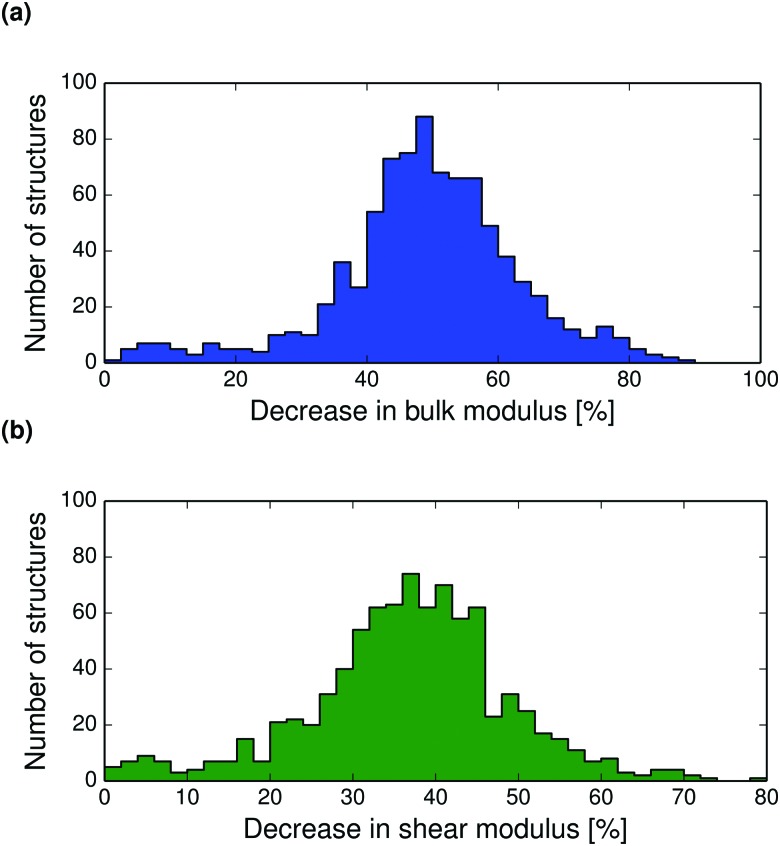
Distributions of the calculated percentage decrease between 0 K and the melting point in (a) the bulk moduli and (b) the shear moduli of all crystal structures in the set.

**Fig. 9:**
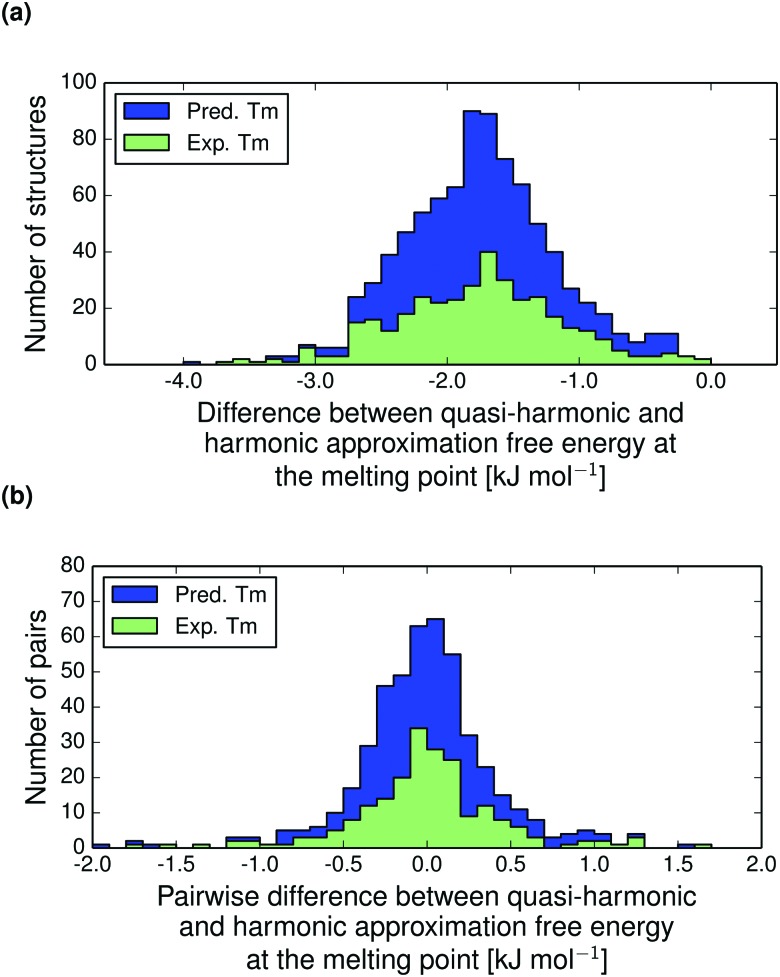
(a) The absolute contribution to the calculated free energy due to thermal expansion for all structures in the polymorph set, calculated at the melting point (*T*_m_) as the difference between the quasi-harmonic (thermally expanded) and harmonic free energy, *A* ^QHA^(*T*_m_) – *A* ^HA^(*T*_*m*_). (b) The pairwise differences in contribution due to thermal expansion to the relative thermodynamic stability of polymorphs at their respective melting temperatures, *Δ*(*A* ^QHA^(*T*_m_) – *A* ^HA^(*T*_m_)). Distributions are shown for molecular crystals with predicted (blue) and experimental (green) melting points.

Our results are consistent with those of Heit and Beran's fragment-based quantum chemical study of four crystals of small molecules (carbon dioxide, water, acetic acid and imidazole), where the neglect of thermal expansion led to free energy errors of up to about 1 kJ mol^–1^.^38^ We find that the effect is often larger than 1 kJ mol^–1^, which is likely due to our set containing larger molecules, for which the absolute energies are larger.

The thermal expansion contributions to the free energy difference between polymorph pairs is shown in Fig. 9b. This distribution is essentially normally distributed around zero with standard deviation 0.42 kJ mol^–1^. Hence, we do not see a trend in the thermal expansion contribution that tends to reduce the free energy difference between polymorphs, as is the case for the harmonic contribution.^14^ The thermal expansion rarely changes the relative stability between polymorphs by more than a small fraction of 1 kJ mol^–1^ and never by more than 2 kJ mol^–1^. For this reason, our harmonic approximation results presented previously^14^ are an accurate description of room temperature polymorph free energy differences.

Thermal expansion also leads to a softening of the crystal structure. The bulk and shear moduli decrease with temperature so that near the melting point, the bulk modulus has reduced by on average 50%, see Fig. 8. The decrease in shear modulus with temperature is slightly less, with most crystals showing a decrease of between 30–50% from 0 K to the melting point. Calculated bulk and shear modulus distributions, and polymorph difference distributions at 298 K are shown in the ESI† (Fig. S27–S30).

**Fig. 10:**
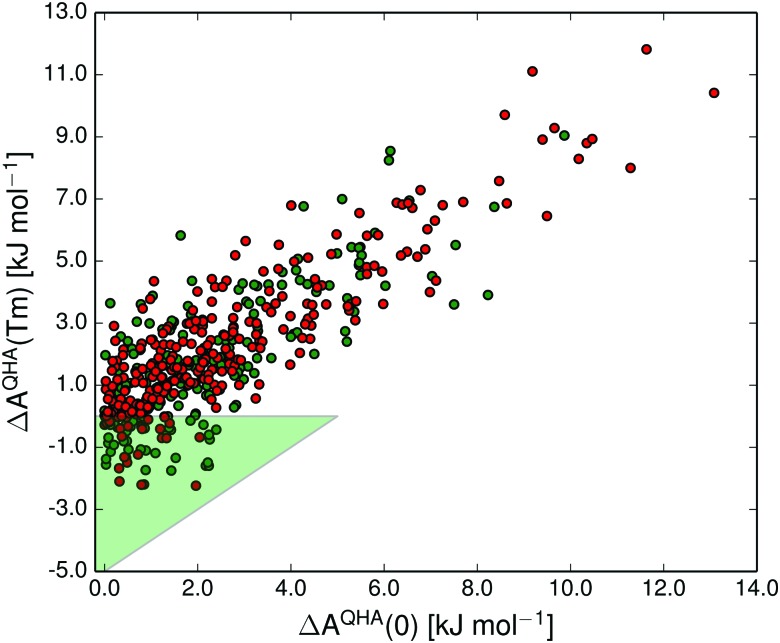
The correlation between the free energy difference in each polymorph pair at 0 K (including vibrational zero-point energy) and the free energy difference at the melting point temperature (*T*_m_) in the quasi-harmonic approximation. The shaded green triangular region marks the 21% of polymorph pairs that were re-ranked by vibrational energy. Green and red data points denote polymorphs for which we have used experimentally measured and predicted melting points, respectively.

In Fig. 10 we show the correlation between polymorph pair-wise energy differences at 0 K (including vibrational zero-point energy) and at the melting point calculated in the quasi-harmonic approximation. Even at the melting temperature, there is a strong correlation between the temperature-dependent free energy and the 0 K energy, just as has been shown previously at room temperature.^14^

**Fig. 11:**
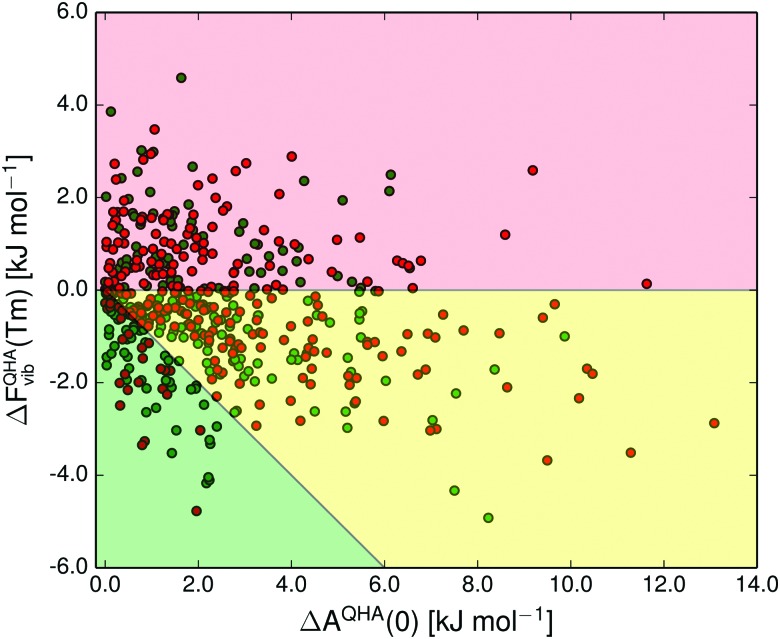
The quasi-harmonic vibrational energy differences at the melting point (*T*_m_) relative to the 0 K relative stability between pairs of polymorphs. The 0 K energies include vibrational zero-point energy. The background colours indicate pairs that have diverging (red) and converging (yellow and green) free energy curves, and which pairs are re-ranked (green). Green and red data points denote polymorphs for which we have used experimentally measured and predicted melting points, respectively.

The shaded green region in Fig. 10 and 11 shows where a re-ranking of the relative thermodynamic stability occurs for the polymorphs between 0 K and the melting point, meaning that the computational model predicts an enantiotropic relationship between polymorphs in this region. In the quasi-harmonic approximation 21% of the pairs fall within this region, compared to 17% in the harmonic approximation. This is substantially more than the 9% we have previously found to be re-ranked below 300 K,^14^ which is expected since the melting points are substantially higher than 300 K for the vast majority of the crystals studied.

The free energy *vs.* temperature curves of polymorphs can either diverge with increasing temperature or converge. Diverging free energy *vs.* temperature curves have previously been described as extremely rare,^12^ because the higher energy polymorph is generally expected to be less well packed, leading to lower vibrational frequencies and a higher entropy. Indeed, only a few polymorph pairs with possibly diverging curves have been studied experimentally.^12,95,96^ Our results, on the other hand, predict that diverging free energy curves should be common, occurring in 38% of cases. At least some of this discrepancy is most probably caused by incorrect lattice energy rankings for some of the polymorphs with our energy model, which might lead to an overestimation of diverging polymorph pairs. For the same reason, the fraction of enantiotropic polymorph pairs (21%) may be underestimated. We have previously shown that trends in polymorph lattice energy and vibrational entropy differences with packing efficiency (or volume) exist, but are very weak.^14^ We believe that the larger proportion of polymorph pairs with diverging free energy *vs.* temperature curves than was previously expected are due to the weakness of these trends and the fact that both lattice energy and entropy have a more complex relationship with crystal structure than simply to packing efficiency. If the prediction in our study is true, it is an important finding that a significant proportion of polymorphic systems have diverging free energy *vs* temperature curves, and this prediction should be revisited as the models for lattice energies and vibrational energy contributions are further improved.

The thermal expansion and the resulting softening of the vibrational modes only marginally affects the relative stabilities of polymorphs. However, since many pairs are just on the border of being re-ranked, even this small contribution is enough to increase the proportion of re-ranked pairs – those whose order of stability changes from 0 K to their melting point – from 17 to 21%. For comparison, we also examined the importance of the zero-point energy by comparing the *A* ^QHA^(*T*_m_) *vs. A* ^QHA^(0) re-ranking (Fig. 10), which includes zero-point energy at 0 K, to the *A* ^QHA^(*T*_m_) *vs. E*_latt_ re-ranking (see ESI,† Fig. S3), where zero-point energy is neglected at 0 K. Neglecting zero-point energy at 0 K changes the proportion of re-ranked structures from 21 to 17%, *i.e.* the same impact as neglecting thermal expansion (Fig. S1, ESI†). These results indicate that the energy changes due to thermal expansion and zero-point energy are of approximately equal importance to polymorph relative stabilities, but both are small compared to the total effect of vibrational energies on polymorph stabilities.

## 4 Conclusions

Quasi-harmonic lattice dynamics calculations have been performed on a large set of polymorphs of organic molecules, as a general assessment of the temperature-dependence of their properties, and to gauge the magnitude of typical property differences between polymorphs. The force field based lattice dynamical methods described here offer a good compromise between accuracy and computational cost, facilitating well-converged lattice-vibrational calculations on hundreds of crystal structures.

The most important observation is that polymorphs have a strong tendency to have very similar properties. Due to compensation between lattice energies and entropies, free energy differences between polymorphs are typically smaller than lattice energy differences, making it very challenging to correctly reproduce temperature dependent polymorph stability rankings. The methods for calculating accurate vibrational energies and entropies are quite well developed, and the errors in calculated static 0 K lattice energy are still an important limitation. Even more accurate force fields are still needed for organic molecular crystals.

Temperature-dependent predictions for individual polymorph systems are still unreliable. However, because of the large set of structures studied here, we are confident in the overall trends. Thus, we predict that at least one fifth of molecular polymorph pairs are enantiotropic. To our knowledge, this is the first prediction of how common enantiotropic polymorphism is in general.

Bulk and shear moduli have been calculated for the entire set of crystal structures. These mechanical properties have a very pronounced temperature-dependence and, while polymorphs tend to have very similar elastic moduli, the differences can be important – we calculate polymorph differences of up to about 2 GPa in the low temperature bulk and shear moduli.

We can reproduce realistic thermal expansions and the temperature-dependence of vibrational spectra, often in good agreement with experimental data.

In crystal structure prediction, harmonic approximation free energies are becoming more commonplace.^33^ Since thermal contributions can be significant, this should improve the predictive ability, and also provide additional insight regarding the properties of the predicted structures. Even when accounting for thermal expansion, lattice vibrations never change the relative stability between polymorphs by more than 5 kJ mol^–1^, at any temperature (Fig. 11). While vibrational energy contributions can, therefore, be important and should not generally be left out of polymorph energy studies, the impact of thermal expansion on the vibrational energy contributions is small, especially when considering free energy differences between polymorphs. Thus, we believe that thermal expansion can safely be ignored for routine CSP-purposes, but should be accounted for when modelling crystal structures at high temperatures, or for benchmarking purposes.^32^

The findings here depend on the reliability of the force field used in the calculations, which does have some systematic errors, such as a small systematic underbinding of crystals. Therefore, we encourage the application of other, perhaps more accurate lattice energy methods for revisiting this large-scale study, as the methods develop and become more computationally accessible. Thus, the entire set of geometry-optimised polymorph pairs – the Nyman Polymorph Library (NPL2016) – is included in the ESI.† This is a large set of polymorph pairs without experimental artefacts such as missing or disordered hydrogen atoms. In addition, we supply melting point temperatures, SMILES and InChi strings for their respective molecules and other data. We hope that this set of crystal structures will facilitate further computational studies of polymorphism.
