# Supplementary material for: Modelling temperature-dependent properties of polymorphic organic molecular crystals
Source: Phys Chem Chem Phys. 2016 Oct 31;18(45):31132–43. doi: 10.1039/c6cp05447a (PMC5299590; doi:10.1039/c6cp05447a)
Supplement: Supplementary file 5 [file CP-018-C6CP05447A-s005.pdf]

Electronic Supplementary Material (ESI) for Physical Chemistry Chemical Physics.
This journal is © the Owner Societies 2016

Supplementary Information for: Modelling temperature-dependent properties of polymorphic organic molecular crystals

Jonas Nyman and Graeme M. Day

1 The Nyman Polymorph Library

We include here additional data that we hope will facilitate future computational studies of polymorph differences.

We provide the optimised crystal structures at both 0 K and at the melting point of each structure in the form of two crystallographic information files (.cif). Please see the files **NPL2016_0K.cif** and **NPL2016_Tm.cif** for these structures. We also provide a list of all Cambridge Structural Database (CSD) reference codes as a txt/gcd. The gcd file can be opened in CSD software (Mercury or Conquest) to view all of the structures.

Additional data is provided in the comma-separated value file (.csv) **NPL2016_data.csv**. This is a text file that can be opened in any text editor or spread sheet program. Each row contains the data for one crystal structure. The data fields are:

- The CSD reference code
- The Standard IUPAC International Chemical Identifier, InChi.
- Z, the number of formula units (molecules) per unit cell
- Z′, the number of formula units per asymmetric unit
- The chemical formula of the molecule.
- The calculated unit cell volume of the 0 K optimised structure, in Å^3
- Experimentally determined melting point temperature in K.
- Predicted melting point temperature in K.
- The thermal pressure at the melting point temperature, in GPa.
- The SMILES string of the molecule.
- The calculated bulk modulus at 0 K in GPa, from the Hill average of the Voigt and Reuss averages over the elastic tensor.
- The calculated shear modulus at 0 K in GPa, from the Hill average of the Voigt and Reuss averages over the elastic tensor.
- The calculated volumetric thermal expansion coefficient in 10^{-6} K^{-1}.

2 Lattice dynamics calculations

Harmonic approximation rigid-body lattice dynamics calculations were performed in DMACRYS 2.0.4,[1] using algorithms that have been described elsewhere.[2–4] Intramolecular vibrations are not considered, and therefore we must limit our investigation to polymorphs with molecules in the same conformation.

Because of the anisotropic phonon dispersion in molecular crystals, it is necessary to consider phonon frequencies calculated at several **k**-points in the first Brillouin zone. We have used our previously described co-prime split linear supercell method[5] for sampling **k**-points, but with a modified co-prime splitting scheme (see ESI†). A target **k**-point separation of 0.1 Å^{-1} was used, resulting in a median of 21 unique **k**-points per structure.

The total number of non-zero phonon frequencies is $n = 6ZN_\mathrm{k} - 3$, where Z is the number of molecules per unit cell and N_k the number of sampled **k**-points. To improve the convergence of vibrational energies with respect to **k**-point sampling, we approximate $g(\omega)$ with a Gaussian kernel density estimate (KDE) of the discrete phonon frequencies ω_i:

$$\frac{g(\omega)}{6Z} \sim \mathrm{KDE}(\omega) = \frac{1}{nh\sqrt{2\pi}} \sum_i^n \exp\left(\frac{-(\omega_i - \omega)^2}{2h^2}\right) \tag{1}$$

In other words, we assume a small dispersion around the sampled **k**-points and replace each phonon frequency by a narrow Gaussian distribution.[4,6] The kernel bandwidth h determines the level of broadening around each calculated frequency, and was set to 1/20 of the standard deviation of the phonon frequencies.

Full elastic tensors were calculated in DMACRYS.[7] The elastic tensor allows us to account for acoustic phonon dispersion near the Γ-point with Debye's method. The Debye frequency ω_D was obtained from the elastic stiffness tensor via the Christoffel equation and the Debye dispersion relation. The Debye frequency for each crystal structure is calculated as the average frequency of acoustic phonons on the surface of an ellipsoid around Γ that extends out to the nearest explicitly sampled **k**-points.

The vibrational contribution to the free energy $F_\mathrm{vib}(T)$ for one unit cell with Z rigid molecules can then be calculated from the phonon density of states $g(\omega)$ and the Debye frequency ω_D as:

$$\begin{aligned} F_\mathrm{vib}(T) = {} & \frac{1}{2}\Big(\frac{n}{n+3}\Big) \int_0^\infty \hbar\omega g(\omega)\ d\omega \\ & + k_B T \Big(\frac{n}{n+3}\Big) \int_0^\infty g(\omega) \ln\left(1 - \exp\left(\frac{-\hbar\omega}{k_B T}\right)\right) d\omega \\ & + \frac{9\hbar\omega_D}{8N_\mathrm{k}} \\ & + \frac{3k_B T}{N_\mathrm{k}} \ln\left(1 - \exp\left(\frac{-\hbar\omega_D}{k_B T}\right)\right) \\ & - \frac{k_B T}{N_\mathrm{k}} \mathrm{D}\Big(\frac{\hbar\omega_D}{k_B T}\Big) \end{aligned} \tag{2}$$

where $\mathrm{D}(x)$ is the Debye function

$$\mathrm{D}(x) = \frac{3}{x^3} \int_0^x \frac{t^3}{\exp(t) - 1}\ dt. \tag{3}$$

The vibrational energy consists of zero point energy ZPE, a thermal contribution to the internal energy and an entropic contribution;

$$F_{\mathrm{vib}}(T) = ZPE + \int_0^T C_{\mathrm{p}}(T)\; dT - TS_{\mathrm{vib}}(T) \tag{4}$$

At non-zero temperatures we calculate the free energy in the harmonic approximation $A^{HA}(T)$ as

$$A^{\mathrm{HA}}(T) = E_{\mathrm{latt}} + F_{\mathrm{vib}}^{\mathrm{HA}}(T) \tag{5}$$

The thermal expansion caused by zero-point vibrations is implicitly included in the empirical force field, which was parametrised to fit lattice energy minima to low temperature crystal structures. Therefore, we calculate the harmonic (HA) and quasi-harmonic (QHA) free energy at 0 K as:

$$A^{\mathrm{QHA}}(0) = A^{\mathrm{HA}}(0) = E_{\mathrm{latt}} + ZPE \tag{6}$$

The harmonic approximation is based on an assumption that phonon frequencies and the crystal structure do not change with temperature. At temperatures near the melting point this leads to unacceptable errors. We have previously described our implementation of a quasi-harmonic method to account for thermal expansion and the temperature-dependence of lattice vibrations.[4]

Thermal expansion and free energies in the quasi-harmonic approximation are calculated using the thermal pressure method.[8] Vibrational contributions to the free energy F_{vib} are calculated for the (0 K) lattice-energy minimised unit cell, and one additional unit cell slightly expanded in volume by the application of a negative hydrostatic pressure. We use -300 MPa, which results in volume expansions of a few percent. We prefer the application of a negative pressure over a direct scaling of lattice parameters because the thermal expansion can be highly anisotropic and because it allows us to calculate elastic tensors and apply the Debye method. A thermal pressure $P_{\mathrm{th}}(T) = -\partial F_{\mathrm{vib}}(T)/\partial V$ is then calculated from a finite difference between the two volumes at a given temperature.

Geometry optimisation in the rigid body approximation at $-P_{\mathrm{th}}$ results in a thermally expanded crystal structure close to the free energy minimum at constant pressure. The quasi-harmonic free energy A^{QHA} can then be calculated for the thermally expanded structure as

$$A^{\mathrm{QHA}}(T) = E_{\mathrm{latt}}(T) + F_{\mathrm{vib}}^{\mathrm{QHA}}(T) \tag{7}$$

3 Included and excluded polymorphs

An alphabetically sorted list of CSD reference codes for all polymorphs included in this study can be found in **NPL2016.gcd**. The file can be opened in CCDC's program Conquest, or as a text file.

The set of polymorphs in this study is based on our previous work, where packing polymorphs were chosen from the 'best hydrogens list' by van de Streek.[9] Not all structures in our previous list could be used however. Below we list some of the reasons we had to exclude structures.

The structures HDXMOR/01, LEZJAB/01, DMANTL01/07 and IFOVOO/01 are conformational polymorphs or have different inter- or intramolecular hydrogen bonding which are not modelled accurately using the methods used in this study, since we neglect intramolecular vibrations.

DMFUSC/04, ACEMID01/03, CATCOL12/13, DMETSO05/06, GLURAC03/04, and YOLDAF01/02 were excluded because they describe the same polymorph and similarly AMBACO09, HACTPH15 and SALOXM05 were excluded because they are re-determinations of AMBACO06, HACTPH11 and SALOXM03, respectively. The high pressure polymorph BENZEN04 was also excluded, since we assume the pressure is zero throughout this study.

The pairs ETDIAM11/16, MAQWIM01/03, PYRENE02/06, QUBPIN/02 and YAMHID/03 are different polymorphs according to CSD data, but converged to the same crystal structure in our geometry-optimisations. These were also excluded, with the exception of ETDIAM11, which forms a pair with ETDIAM18, which is a distinct polymorph.

Disordered structures (DOGWOL01) and structures with missing hydrogen atoms (MEQVAG/01, AMYTAL10/11, PMAANO01/10, PTCDEC/01 and HUMTEP01) were also excluded.

For the families MSULIN and HUPGOO the generated SMILES could not be used in MTBTNT, so no predicted melting points could be obtained.

During CRYSTALOPTIMIZER calculations, structures of the families BDTOLE, NUQXEC, BIRKIW, PEWXAQ, SAXJEG, UTORAX, YIVRIF, JEJSIA and FUGYAH had to be excluded because of convergence issues in calculating the molecular Hessian matrix. The size or shape of some systems (BIPHME, MOHDET, HOFMAQ, UNOGIN, AWIFUI, DAVWAY, FIMNAQ, HPTHEL, MATDEQ, IDALEF and YIHKAB) also made convergence difficult.

For extremely anisotropic unit cells, the method of **k**-point sampling in the lattice dynamics calculations we use can be computationally cumbersome. The structures BANHOO/01 and CBFBZF01 had to be excluded because we could not sample the required **k**-points.

4 Structures used in thermal expansion coefficient calculations

The following structures, referred to by their Cambridge Structural Database reference codes, were used for calculating the experimental values of volumetric thermal expansion coefficients.

monoclinic MNPHOL: MNPHOL02; MNPHOL10; MNPHOL11; MNPHOL12; MNPHOL13; MNPHOL14; MNPHOL15; MNPHOL16; MNPHOL17; MNPHOL26.

1,2-ethanediamine (ETDIAM) form I alpha; ETDIAM01; ETDIAM10; ETDIAM11; ETDIAM12; ETDIAM13.

beta-sulfur (FURHUV): FURHUV03; FURHUV04; FURHUV05; FURHUV06; FURHUV07; FURHUV08; FURHUV10.

glutaric acid (GLURAC) beta form: GLURAC07; GLURAC08; GLURAC09; GLURAC10; GLURAC11; GLURAC12.

paracetamol (HXACAN) form I: HXACAN01; HXACAN02; HXACAN03; HXACAN04; HXACAN05; HXACAN06; HXACAN07; HXACAN13; HXACAN14; HXACAN15; HXACAN16; HXACAN17; HXACAN18; HXACAN19; HXACAN20; HXACAN26; HXACAN27; HXACAN28; HXACAN30; HXACAN34.

adipic acid (ADIPAC) form I: ADIPAC; ADIPAC01; ADIPAC02; ADIPAC04; ADIPAC06; ADIPAC07; ADIPAC08; ADIPAC09; ADIPAC11; ADIPAC13; ADIPAC14; ADIPAC15; ADIPAC16; ADIPAC17; ADIPAC18; ADIPAC19.

2,2'-Dipyridylamine (DPYRAM) monoclinic form: DPYRAM02 and DPYRAM03.

Pyrazinamide (PYRZIN) beta form: PYRZIN01; PYRZIN18 and PYRZIN23.

Structures whose temperature is reported as room temperature were treated as 293 K.

5 Additional results

Below are figures with additional results that could not be included in the main article.

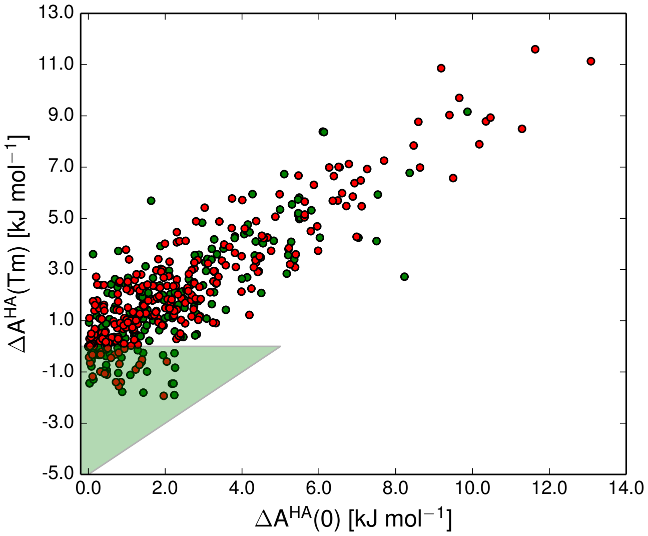

Figure S1: The correlation between the free energy difference in each polymorph pair at 0 K and the free energy difference at the melting point in the harmonic approximation. The green triangle marks the 17% of pairs that were re-ranked by vibrational energy. Green and red data points represent experimentally determined and predicted melting points respectively.

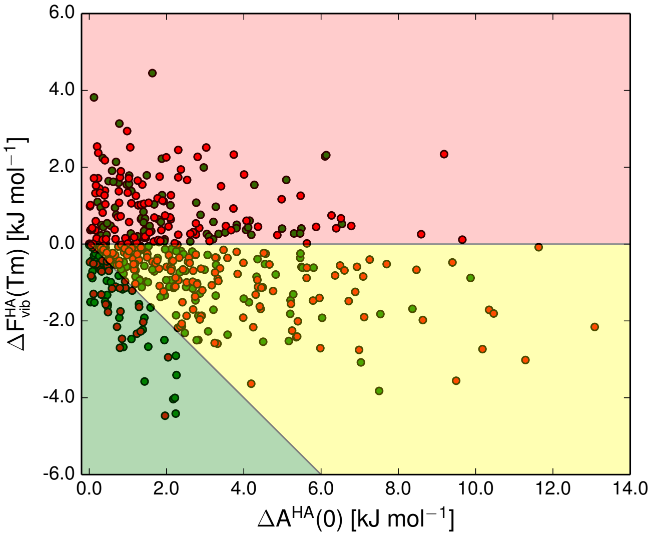

Figure S2: The harmonic approximation vibrational energy differences at the melting point relative to the 0 K relative stability between pairs of polymorphs. The background colours indicate pairs that have diverging (red) and converging (yellow and green) free energy curves, and which pairs are re-ranked (green). Green and red data points represent experimentally determined and predicted melting points respectively.

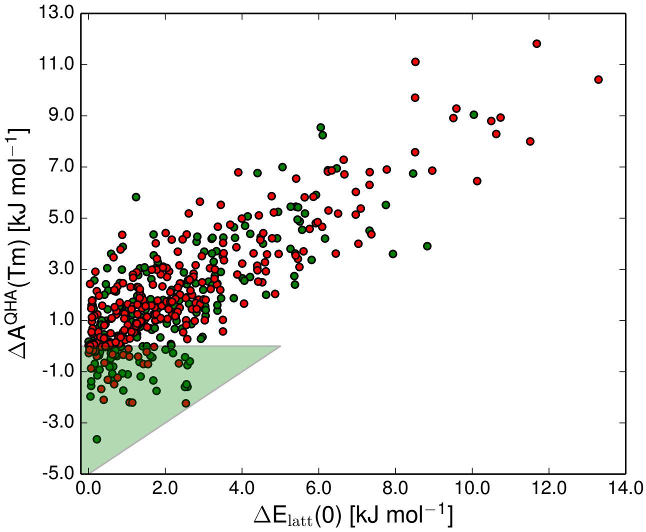

Figure S3: The correlation between the 0 K lattice energy and the quasi-harmonic free energy at the respective melting points. The correlation coefficient is 0.79, meaning that lattice energy differences accounts for 79% of free energy differences at high temperatures. The fraction of pairs that are re-ranked by free energy relative to the lattice energy ranking (green field) is 17%.

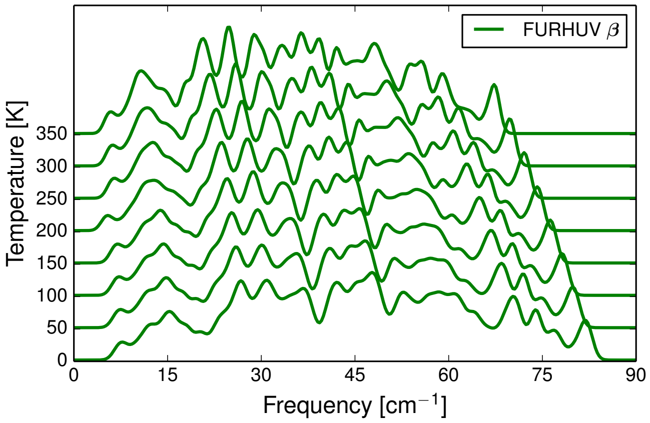

Figure S4: Phonon densities of state at different temperatures for β-sulphur (FURHUV10).

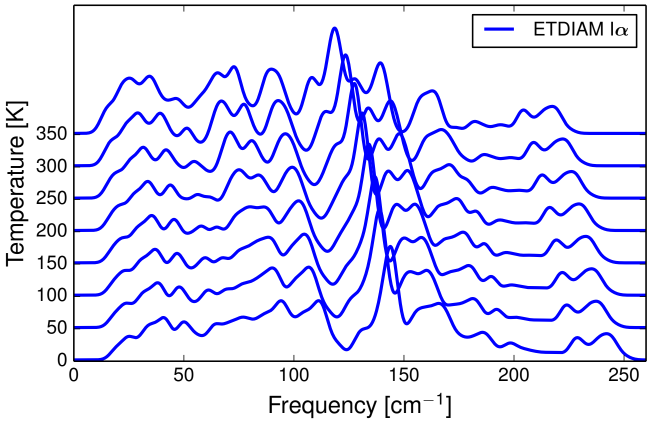

Figure S5: Phonon densities of state at different temperatures for 1,2-ethanediamine (ETDIAM11).

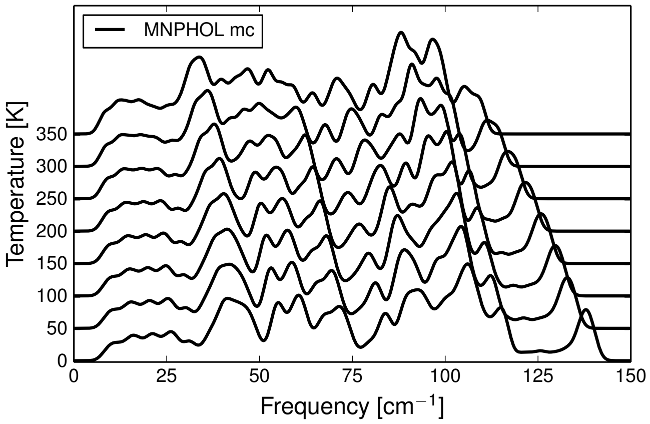

Figure S6: Phonon densities of state at different temperatures for m-nitrophenol (MNPHOL02).

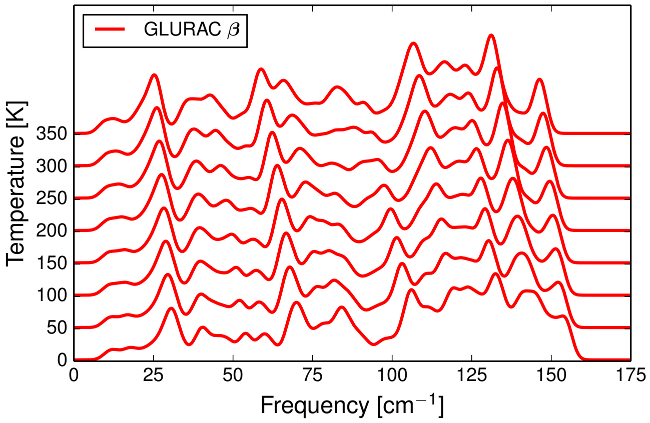

Figure S7: Phonon densities of state at different temperatures for glutaric acid β (GLURAC03).

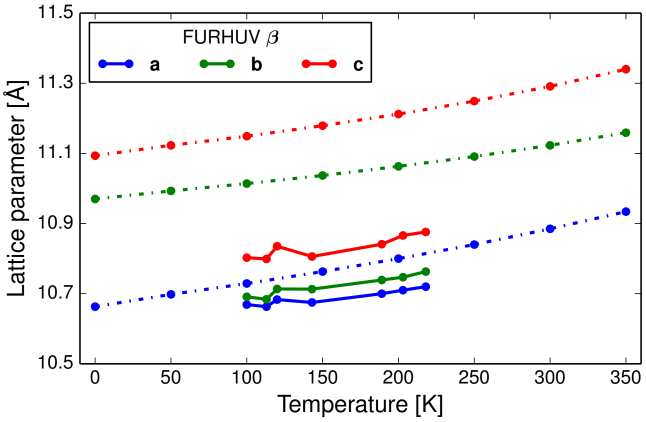

Figure S8: Calculated thermal expansion (dashed line) of the β sulfur polymorph (FURHUV10) compared to experimental data (solid line).

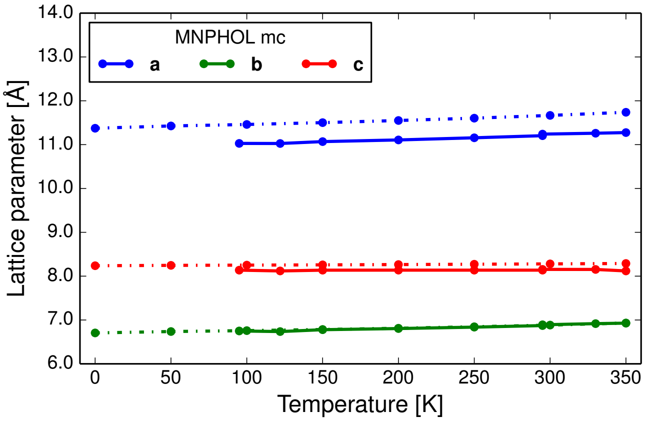

Figure S9: Calculated thermal expansion (dashed line) of monoclinic m-nitrophenol (MNPHOL02) compared to experimental data (solid line).

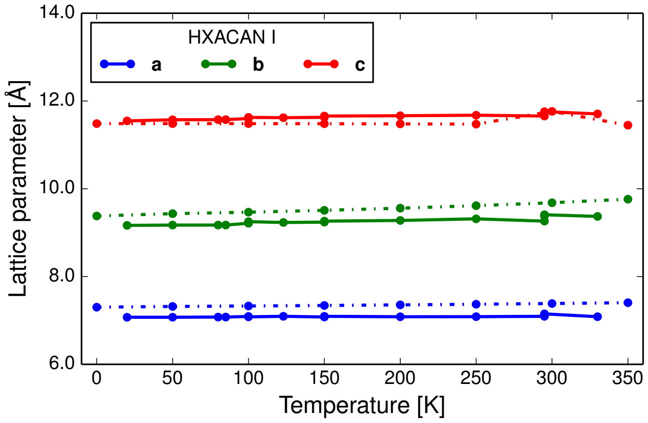

Figure S10: Calculated thermal expansion (dashed line) of paracetamol form I (HXACAN12) compared to experimental data (solid line).

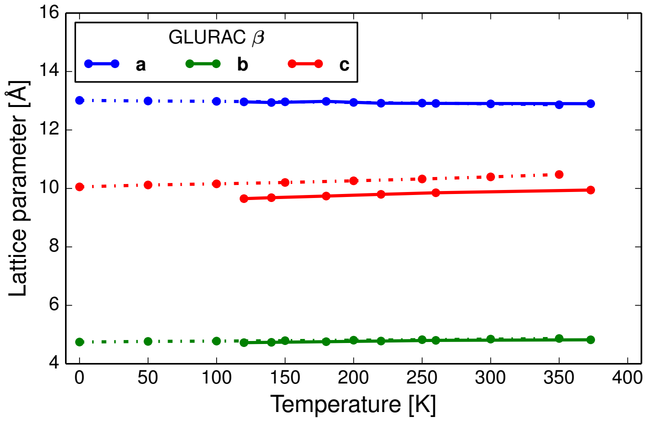

Figure S11: Calculated thermal expansion (dashed line) of the β glutaric acid polymorph (GLURAC03) compared to experimental data (solid line).

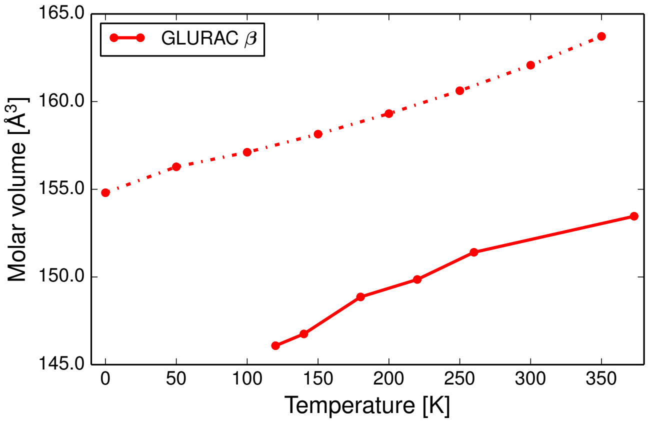

Figure S12: Calculated volumetric thermal expansion (dashed line) of the β glutaric acid polymorph (GLURAC03) compared to experimental data (solid line).

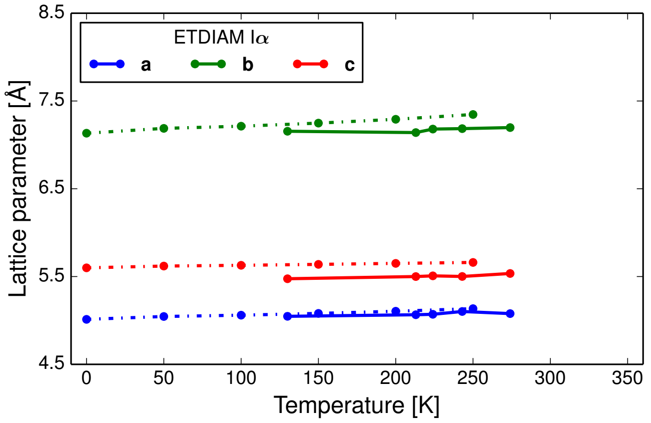

Figure S13: Calculated thermal expansion (dashed line) of lattice vectors of the Iα 1,2-ethanediamine polymorph (ETDIAM11) compared to experimental data (solid line).

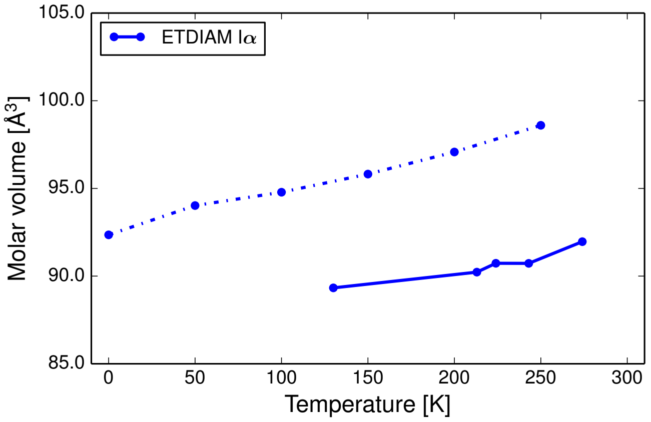

Figure S14: Calculated volumetric thermal expansion (dashed line) of the Iα 1,2-ethanediamine polymorph (ETDIAM11) compared to experimental data (solid line).

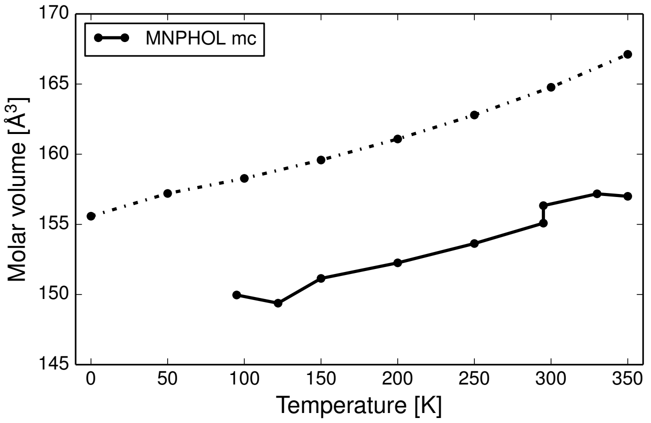

Figure S15: Calculated volumetric thermal expansion (dashed line) of monoclinic m-nitrophenol (MNPHOL02) compared to experimental data (solid line).

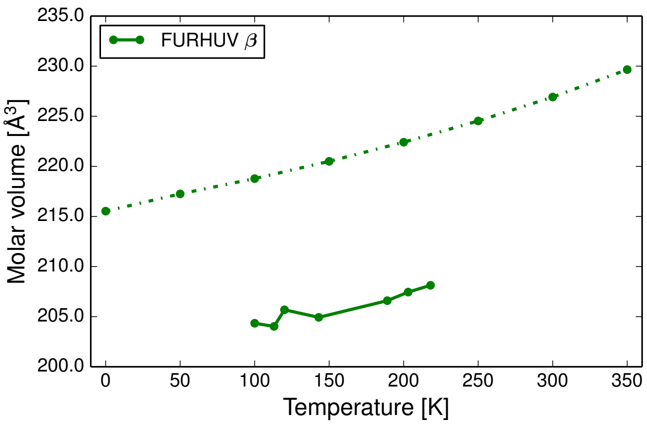

Figure S16: Calculated volumetric thermal expansion (dashed line) of β sulfur (FURHUV12) compared to experimental data (solid line).

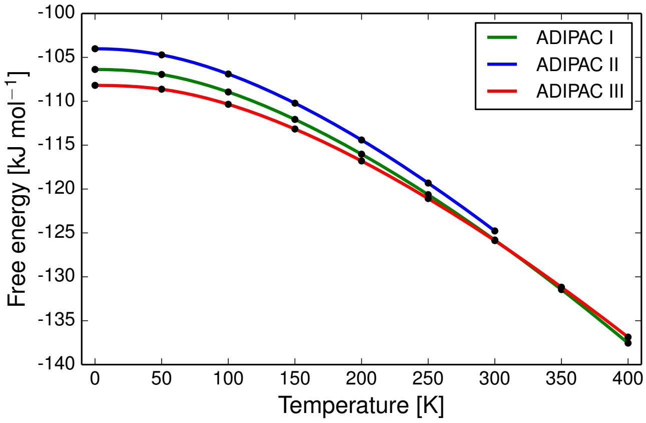

Figure S17: Quasi-harmonic free energy curves for polymorphs of adipic acid.

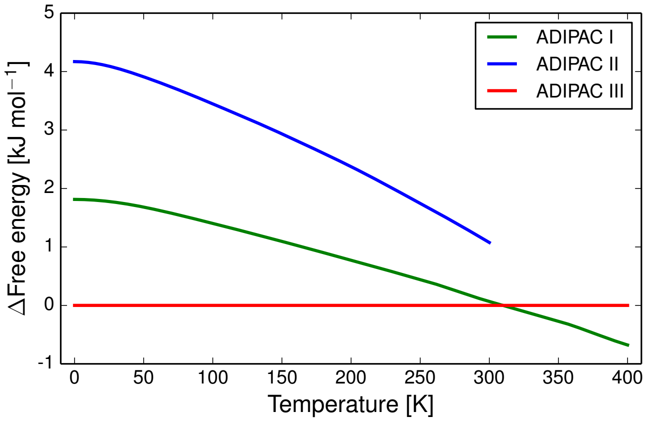

Figure S18: Quasi-harmonic free energy difference curves for polymorphs of adipic acid.

Adipic acid (1,6-hexanedioic acid) has three experimentally known polymorphs.[10,11] There is a phase transition between forms I and II at 130-136 K. It is first-order, enantiotropic and reversible. Form I is thermodynamically stable at room temperature, II is stable below 130 K. Computationally, form II became unstable at high temperatures (Born instability). No transitions have been described in the triclinic phase III and it appears to be monotropic relative to the other forms.

Our calculated free energy curves for adipic acid are shown in Figures S15 and S16. We predict form III to be more stable than the others below 310 K, a possibility which strictly is not ruled out by experimental data. The calculated free energy curves are incorrect because of the 0 K lattice energy ordering of the polymorphs.

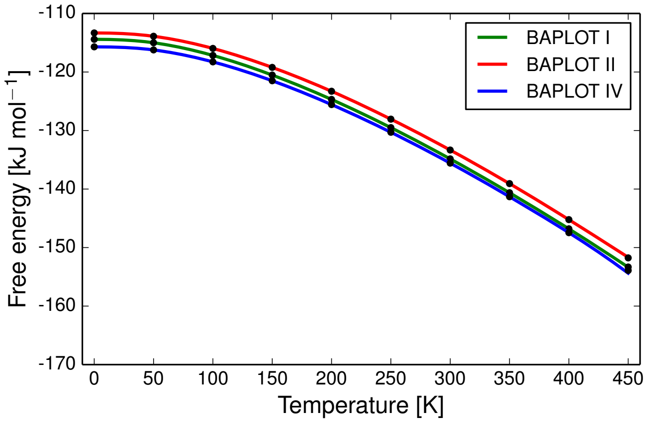

Figure S19: Quasi-harmonic free energy curves for polymorphs of theophylline. Polymorph IV changes structure slightly above 450 K, becoming less stable.

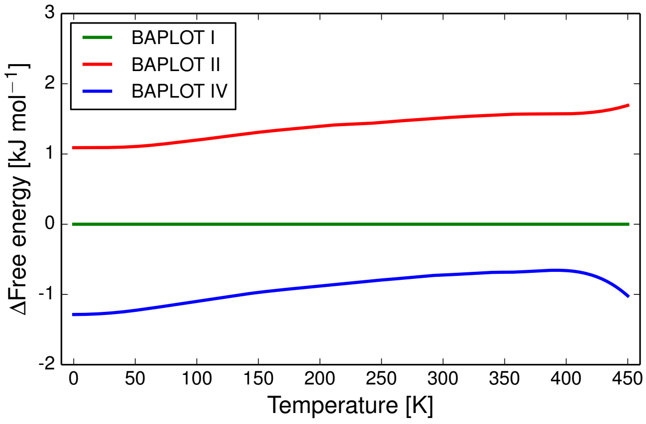

Figure S20: Quasi-harmonic free energy difference curves for polymorphs of theophylline. Polymorph IV changes structure slightly above 450 K, becoming less stable.

Figures S19 and S20 show the relative stabilities of three polymorphs of theophylline (CSD BAPLOT). Form I is a high-temperature polymorph and is enantiotropically related to form II. Form II was believed to be the thermodynamically most stable form at ambient conditions until form IV was discovered. There is a (monotropic) transition from IV to II between 483 and 513 K.[12] The structure of form IV becomes unstable around 450 K in the quasi-harmonic approximation.

The experimental data suggests form IV is thermodynamically stable between 295 and 483 K. Form II is then the most stable between 513 and 536, and form I is favoured up to the melting point. Our calculated free energy curves for theophylline I, II and IV are shown in Figures S17 and S18. We correctly identify from IV as the most stable at low temperatures, and that form I has a higher entropy than the other structures. Form I is however incorrectly found to be monotropically related to form II. Note the very small energy differences between these polymorphs, which are smaller than the expected errors in the energy model.

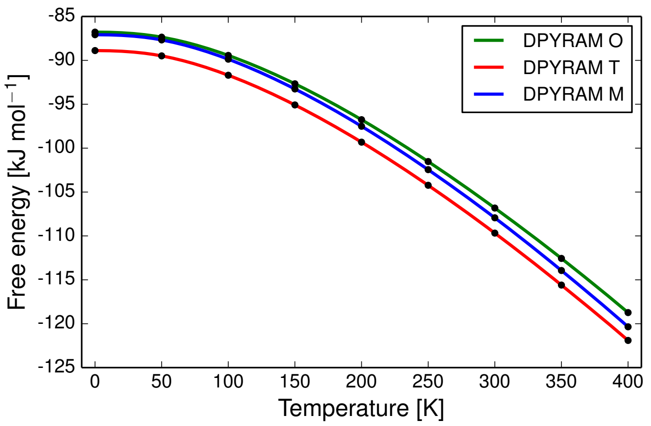

Figure S21: Quasi-harmonic free energy curves for polymorphs of 2,2′-dipyridylamine.

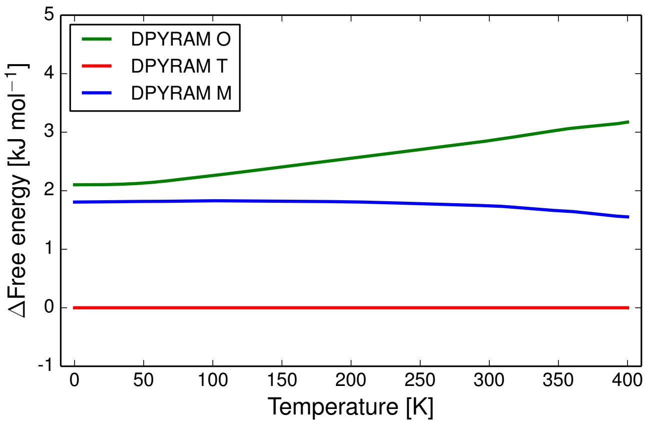

Figure S22: Quasi-harmonic free energy difference curves for polymorphs of 2,2′-dipyridylamine.

The curves in Figures S21 and S22 show the calculate relative thermodynamic relationships between the orthorhombic (DPYRAM), triclinic (DPYRAM01) and monoclinic (DPYRAM02) polymorphs of 2,2′-dipyridylamine. These can be compared to qualitative curves based on experimental data.[13]

The orthorhombic polymorph should be unstable at high temperatures, so the fact that this is the least entropically stabilized form is correct. However, it should be the most stable form below 263 K, indicating that our lattice energy ranking of the polymorphs is incorrect. The stability reordering between the triclinic and monoclinic forms at 310 K is not reproduced, because we incorrectly predict the triclinic form to the thermodynamically stable form at room temperature, rather than the monoclinic form. Thus, the relative entropies seem to be accurate, but the static energy differences between the three polymorphs are in error.

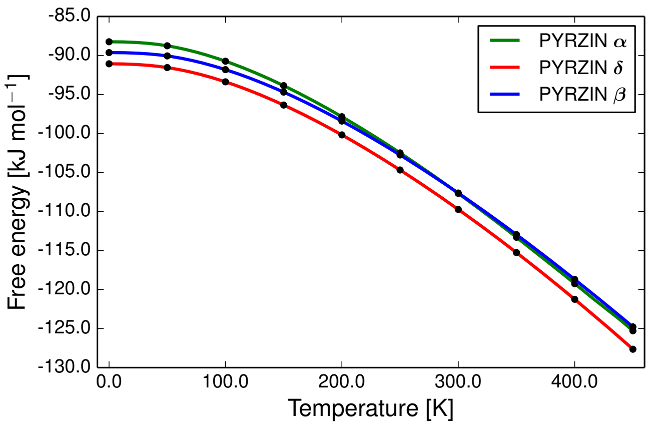

Figure S23: Quasi-harmonic free energy curves for polymorphs of pyrazine-2-carboxamide.

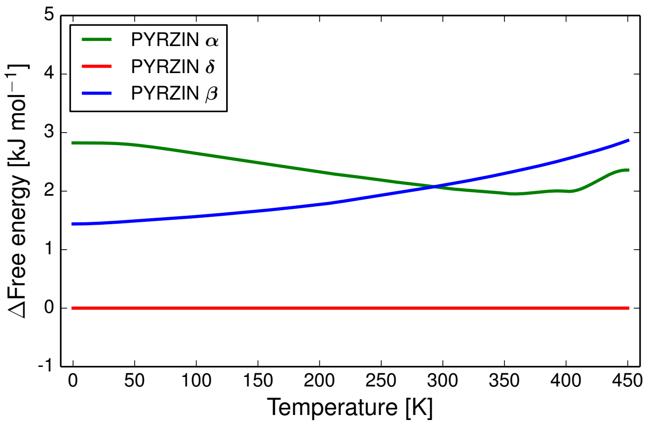

Figure S24: Quasi-harmonic free energy difference curves for polymorphs of pyrazine-2-carboxamide.

For pyrazine-2-carboxamide, (pyrazinamide, rifater) we have used three of the known polymorphs. The different forms are denoted by Greek letters; α, β and δ corresponding to CSD structures PYRZIN15, PYRZIN18 and PYRZIN16, respectively.[14]

Experimental results indicate that the order of stability is $\delta < \alpha < \beta$ at 0 K, with δ being the most stable.[15] At elevated temperatures, the order is $\alpha < \delta < \beta$.[16] A semi-quantitative free energy diagram has been constructed based on experimental data.[14] We correclty predict the δ form as the most stable, but the calculated 0 K order of α and β forms is incorrect. The α is correctly predicted to approach δ with increasing temperature, but the calculated free energy curves do not cross.

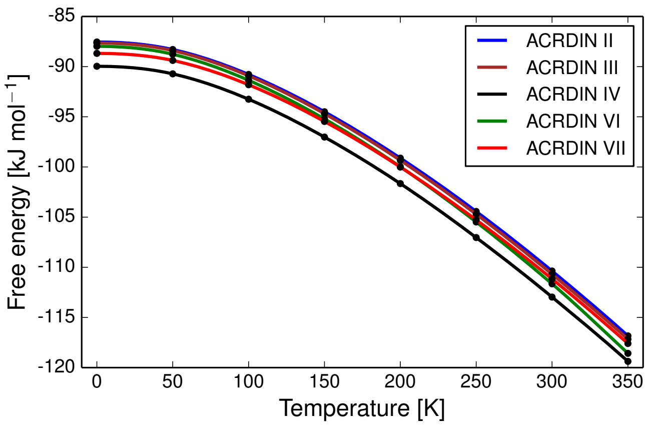

Figure S25: Quasi-harmonic free energy curves for several polymorphs of acridine (ACRDIN).

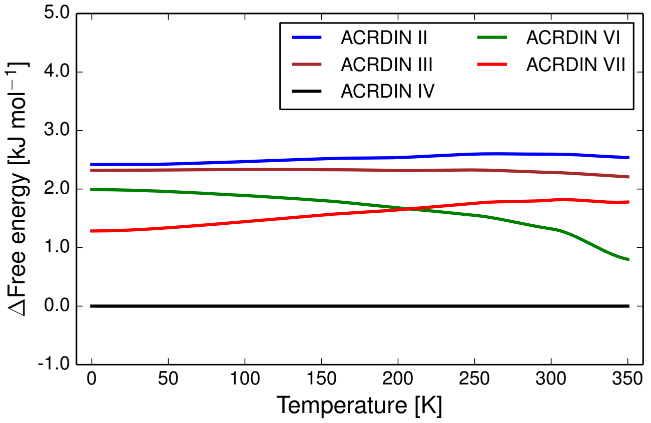

Figure S26: Quasi-harmonic free energy difference curves for several polymorphs of acridine, relative to polymorph IV

In acridine, the relative stabilities between the many known forms can be inferred from melting points,[17], slurry experiments[18] and known phase transitions. Form II was originally reported as the thermodynamically stable form at ambient conditions,[19] but slurry experiments suggest form III to be thermodynamically favoured at 295 K, and that it converts to form II at 318 K.[19] Forms II and IV can crystallize concomitantly,[17] yet form IV converts to II at 348 K.[19] Phase II then converts enantiotropically to form VIII at 374 K.[17] The available data shows that all polymorphs are very similar in enthalpy, but that possibly the following orders of stability can be assigned at low temperatures, III>II>IV, and high, VIII>VII>VI>IV. The calculated curves do not quantitatively agree with experimental data, see Figs S25 and S26. The lattice energy ordering is incorrect and hence, the free energy curves are necessarily also incorrect.

We have previously published results on the thermodynamics of polymorphs at room temperature,[5] but elastic properties were not considered in that study. Since the elastic properties are highly temperature dependent, we include here additional results on the distributions of absolute and relative differences in shear and bulk moduli at room temperature, see Figs S27 – S30. Results are presented for a subset of 801 crystal structures with melting points above room temperature, resulting in 444 pairwise comparisons.

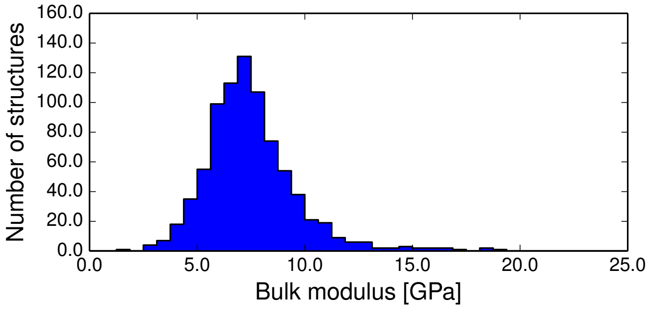

Figure S27: The distribution of bulk moduli calculated at 298 K.

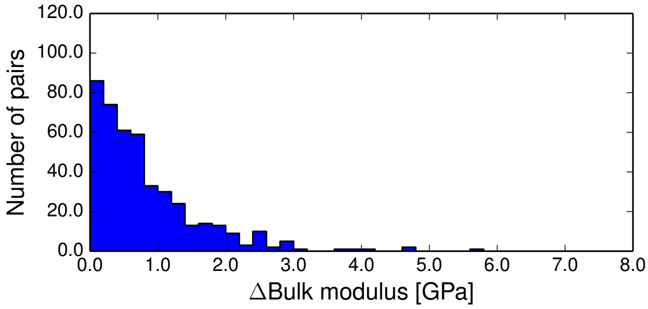

Figure S28: The distribution of pairwise differences in bulk moduli between 444 polymorph pairs calculated at 298 K.

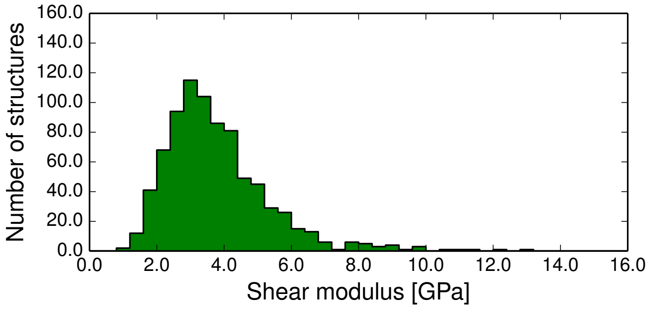

Figure S29: The distribution of shear moduli calculated at 298 K.

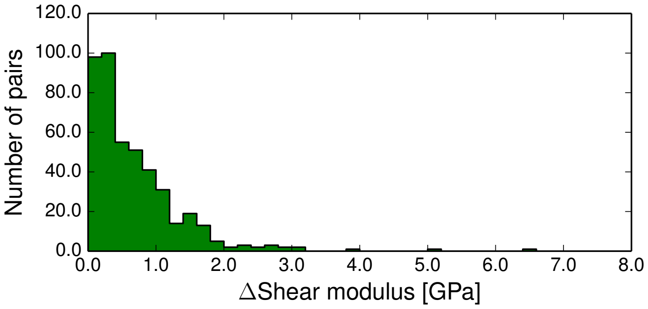

Figure S30: The distribution of pairwise differences in shear moduli between 444 polymorph pairs calculated at 298 K.

6 Flexibility rules

A python script was used to automatically select the most relevant flexible degrees of freedom from the Z-matrix generated by CrystalOptimizer. The algorithm used for constructing the Z-matrix is described in Appendix A of the PhD-thesis by Kazantsev.[20] Our script analyses the atomic connectivity in the molecule by means of standard graph-theoretical algorithms[21] and chooses degrees of freedom according to these rules:

- Covalent bond lengths are optimised without considering packing forces;
- All angles and dihedrals containing a polar hydrogen atom (–OH, –NH, –SH[1]) are optimised under the influence of packing forces;
- All exocyclic bonds are considered rotatable and are optimised under the influence of crystal packing forces;
- Dihedrals and angles in 3- and 4-membered rings are optimised without considering packing forces;
- Dihedrals and angles in 5- and 6-membered rings consisting of 3-coordinated carbon atoms and nitrogen in any combination are unaffected by packing forces, except dihedrals and angles that contain a polar hydrogen atom;
- Dihedrals and angles in 5-membered rings containing sulfur or oxygen[2] atoms bonded to two 3-coordinated carbon atoms are optimised without considering packing forces;
- Any remaining dihedrals are optimised with respect to packing forces;
- Linear angles in –C≡N and –C≡C– groups are constrained to 179.99°.

For completely rigid molecules we allow the first angle to be optimized with respect to packing forces, since CrystalOptimizer needs at least one flexible degree of freedom. We use CrystalOptimizer also on formally rigid molecules in order to optimise them to the minimum DFT energy conformer. In particular this optimises the hydrogen positions in a consistent way.

[1]There are no thiols in the structure set, as very few polymorphic thiol compounds are known. Also, the force field used in this work does not contain parameters for thiol hydrogen atoms.

[2]Specifically furan, thiophene, 1,3-oxazole, 1,3-thiazole, 1,3,4-oxadiazole and 1,3,4-thiadiazole rings.

7 Regression analysis for melting point vs. lattice energy

We expect the melting temperature to depend simply on the enthalpy/lattice energy. Since we have a good anisotropic model potential to calculate accurate lattice energies, trying to predict melting points based on this lattice energy is attractive. Here we have used only the intermolecular part of the lattice energy. Intramolecular contributions arising from geometric distortions from the vacuum conformation are not included here for simplicity.

To obtain an adequate regression model, it is necessary to perform a variance stabilizing transformation. Assuming the data is Poisson-distributed, the variance can be made homoscedastic with the Anscombe transform:

$$x \mapsto 2\sqrt{x + 3/8} \tag{8}$$

In this case the 3/8 term is small and simply taking the square root of the lattice energies is adequate and gives virtually identical results.

We used Minitab 17 to perform the least squares regression. The full output is provided below. The residuals for the resulting model (Fig. S32) have a standard deviation of 54.5 K, the residuals are normally distributed, independent and homoscedastic, giving us confidence in the model.

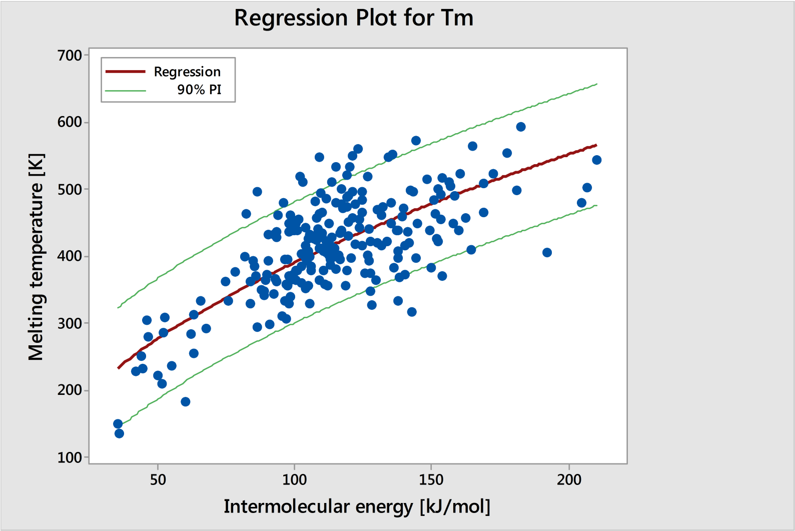

Figure S31: Experimental melting points vs. intermolecular energies. Linear fit (red line) and the non-linear regression (yellow line) use here.

```
Starting Values for Parameters
Parameter  Value
Theta1        37
Theta2      0.5*
* Locked.

Equation
Tm = 38.9938 * 'Lattice energy' ^ 0.5

Parameter Estimates
```

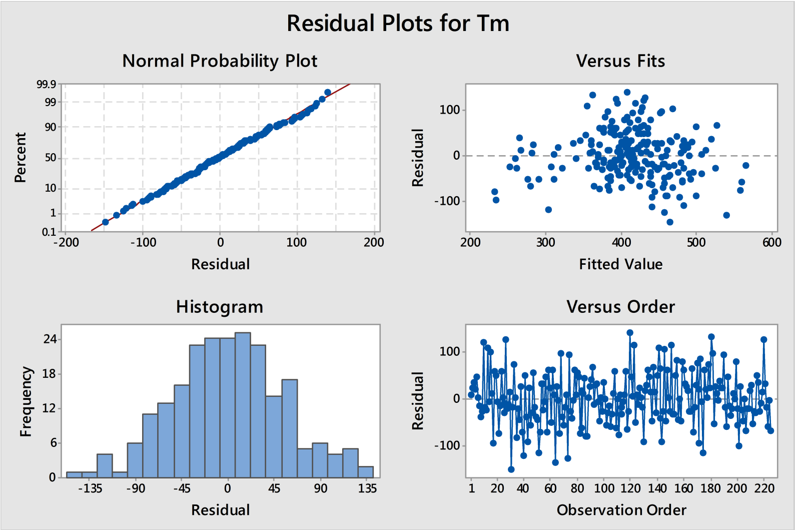

Figure S32: Residual analysis for the regression model.

```
Parameter  Estimate  SE Estimate
Theta1      38.9938     0.339152
Theta2       0.5000            *

Tm = Theta1 * 'Lattice energy' ^ Theta2

Lack of Fit
Source           DF      SS       MS     F      P
Error           224  665027  2968.87
  Lack of Fit   222  660987  2977.42  1.47  0.492
  Pure Error      2    4040  2020.00

Summary
Iterations          2
Final SSE      665027
DFE               224
MSE           2968.87
S             54.4873
```

8 Co-prime splitting of linear supercells.

The crystal unit cell is expanded into linear supercells expanded along **a**, **b** or **c** only. The co-prime splitting used in this article differs from that used in our previous paper[5]. We have changed the co-prime splitting for two reasons. Firstly, we want to make the convergence less erratic and more monotonic. This is achieved by making sure that as the target k-point distance decreases, the number of sampled k-points increase strictly. Because of a minor error in our previous method, this was not strictly guaranteed.

The co-prime splitting used in this article is as follows. If $s < 6$ the lattice dynamic calculations is performed on the supercell as is. For $s \geqslant 6$ the supercell is split into n smaller supercells (1x1xk, 1x1xℓ, 1x1xm ...) such that $k, \ell, m...$ are all mutually co-prime and $k + \ell + m - n \geqslant s$. This ensures that at least the same number of **unique k**-points are sampled. The phonons for $\mathbf{k} = \mathbf{0}$ will be calculated in each supercell, and we only include these phonons from one of the split supercells. The long linear supercells are split into 2, 3 or 4 co-prime supercells according to the scheme in Table S1. Note that this is by no means the only possible choice, and we make no claim that this is the best or computationally most efficient splitting. Splitting the supercells means that the sampled **k**-points will no longer be equidistantly placed along the reciprocal axes, but this has a very small effect on the results.

Table S1: Linear supercells were split into 2, 3 or 4 smaller supercells with mutually co-prime expansion coefficients in this way.

2	→	2
3	→	3
4	→	4
5	→	5
6	→	3, 4
7	→	3, 5
8	→	4, 5
9	→	3, 4, 5
10	→	3, 4, 5
11	→	3, 4, 7
12	→	3, 4, 7
13	→	3, 5, 7
14	→	4, 5, 7
15	→	5, 6, 7
16	→	3, 4, 5, 7
17	→	5, 7, 8
18	→	5, 7, 8
19	→	3, 5, 7, 8
20	→	3, 5, 7, 8
21	→	4, 5, 7, 9
22	→	4, 5, 7, 9
23	→	4, 5, 7, 9
24	→	5, 7, 8, 9
25	→	5, 7, 8, 9
26	→	5, 7, 8, 9

9 Model potential parameters for halogens

Halogen atoms tend to have an anisotropic van der Waals radius[22]. To account for this, intermolecular potentials with an anisotropic repulsion term have been developed[1,23]. A local unit vector $\mathbf{e}_z$ is defined at each anisotropic site, parallel to the covalent bond joining the halogen to its bonded atom, pointing away from the bond. A second unit vector, $\mathbf{e}_{ik}$, is the vector between the interacting atoms. DMACRYS describes repulsion anisotropy using a modified exp-6 potential of the form:

$$V = G \exp\left(-B^{\iota\kappa}(r_{ij} - \rho^{\iota\kappa}(\Omega_{ik}))\right) - C^{\iota\kappa}/r^6, \tag{9}$$

where $\rho^{\iota\kappa}(\Omega_{ik})$ describes the anisotropy of repulsion, and is defined as:

$$\begin{aligned}\rho^{\iota\kappa}(\Omega_{ik}) = \rho_0^{\iota\kappa} + \rho_1^{\iota}(\mathbf{e}_z^i \cdot \mathbf{e}_{ik}) + \rho_1^{\kappa}(-\mathbf{e}_z^k \cdot \mathbf{e}_{ik}) + \rho_2^{\iota}(3[\mathbf{e}_z^i \cdot \mathbf{e}_{ik}]^2 - 1)/2 \\ + \rho_2^{\kappa}(3[\mathbf{e}_z^k \cdot \mathbf{e}_{ik}]^2 - 1)/2\end{aligned} \tag{10}$$

ρ_0 describes the isotropic repulsion, ρ_1 parameters describe a shift of the centre of repulsion and ρ_2 parameters describe a quadrupolar distortion of the atom. Parameters for Cl and F were taken from Day's specifically developed potential for molecule XIII in the 4th blind test of crystal structure prediction.[24] Halogen parameters were empirically fitted to reproduce the crystal structures of a set of halogenated aromatic molecules. Details are available in the ESI to the 4th blind test paper. The parameters, in input format for DMACRYS are provided below:

```
BUCK   F_01  F_01
   3761.006673    0.240385    7.144500   0.0  70.0
ANIS   F_01  F_01
 0 0 2 0 2 -0.035000
 0 0 0 2 2 -0.035000
ENDS
BUCK   Cl01  Cl01
   5903.747391    0.299155   86.716330   0.0  70.0
ANIS   Cl01  Cl01
 0 0 2 0 2 -0.093860
 0 0 0 2 2 -0.093860
ENDS
```

Heteroatomic interactions between sulfur, fluorine and chlorine and other atom types are calculated using conventional arithmetic and geometric averages of the force field parameters:

$$A^{\iota\kappa} = \sqrt{A^{\iota\iota}A^{\kappa\kappa}} \tag{11}$$

$$B^{\iota\kappa} = \frac{B^{\iota\iota}B^{\kappa\kappa}}{2} \tag{12}$$

$$C^{\iota\kappa} = \sqrt{C^{\iota\iota}C^{\kappa\kappa}} \tag{13}$$

References

[1] S. L. Price, M. Leslie, G. W. A. Welch, M. Habgood, L. S. Price, P. G. Karamertzanis and G. M. Day, *Phys. Chem. Chem. Phys.*, 2010, **12**, 8478–8490.

[2] G. M. Day, S. L. Price and M. Leslie, *The Journal of Physical Chemistry B*, 2003, **107**, 10919–10933.

[3] N. Neto, R. Righini, S. Califano and S. Walmsley, *Chemical Physics*, 1978, **29**, 167–179.

[4] J. Nyman, O. S. Pundyke and G. M. Day, *Phys. Chem. Chem. Phys.*, 2016, **18**, 15828–15837.

[5] J. Nyman and G. M. Day, *CrystEngComm*, 2015, **17**, 5154–5165.

[6] A. M. Karo and J. R. Hardy, *Physical Review*, 1969, **181**, 1272.

[7] G. M. Day, S. L. Price and M. Leslie, *Crystal Growth & Design*, 2001, **1**, 13–27.

[8] A. Otero-de-la-Roza and E. R. Johnson, *The Journal of chemical physics*, 2012, **137**, 054103.

[9] J. van de Streek, *Acta Crystallographica Section B*, 2006, **62**, 567–579.

[10] R. S. Gopalan, P. Kumaradhas and G. Kulkarni, *Journal of Solid State Chemistry*, 1999, **148**, 129–134.

[11] H.-K. Fun and S. Chantrapromma, *Acta Crystallographica Section E*, 2009, **65**, o624.

[12] D. Khamar, I. J. Bradshaw, G. A. Hutcheon and L. Seton, *Crystal Growth & Design*, 2011, **12**, 109–118.

[13] H. Schödel, C. Näther, H. Bock and F. Butenschön, *Acta Crystallographica Section B*, 1996, **52**, 842–853.

[14] S. Cherukuvada, R. Thakuria and A. Nangia, *Crystal Growth & Design*, 2010, **10**, 3931–3941.

[15] A. Borba, M. Albrecht, A. Gómez-Zavaglia, M. A. Suhm and R. Fausto, *The Journal of Physical Chemistry A*, 2009, **114**, 151–161.

[16] R. A. Castro, T. M. Maria, A. O. Évora, J. C. Feiteira, M. R. Silva, A. M. Beja, J. Canotilho and M. E. S. Eusébio, *Crystal Growth & Design*, 2009, **10**, 274–282.

[17] D. Braga, F. Grepioni, L. Maini, P. P. Mazzeo and K. Rubini, *Thermochimica Acta*, 2010, **507-508**, 1–8.

[18] D. Musumeci, C. A. Hunter and J. F. McCabe, *Crystal Growth & Design*, 2010, **10**, 1661–1664.

[19] B. P. Clarke, J. M. Thomas and J. O. Williams, *Chemical Physics Letters*, 1975, **35**, 251–254.

[20] A. Kazantsev, *Ph.D. thesis*, Imperial College London, 2011.

[21] A. A. Hagberg, D. A. Schult and P. J. Swart, Proceedings of the 7th Python in Science Conference (SciPy2008), Pasadena, CA USA, 2008, pp. 11–15.

[22] S. C. Nyburg and C. H. Faerman, *Acta Crystallographica Section B*, 1985, **41**, 274–279.

[23] G. M. Day and S. L. Price, *Journal of the American Chemical Society*, 2003, **125**, 16434–16443.

[24] G. M. Day, T. G. Cooper, A. J. Cruz-Cabeza, K. E. Hejczyk, H. L. Ammon, S. X. M. Boerrigter, J. S. Tan, R. G. Della Valle, E. Venuti, J. Jose, S. R. Gadre, G. R. Desiraju, T. S. Thakur, B. P. van Eijck, J. C. Facelli, V. E. Bazterra, M. B. Ferraro, D. W. M. Hofmann, M. A. Neumann, F. J. J. Leusen, J. Kendrick, S. L. Price, A. J. Misquitta, P. G. Karamertzanis, G. W. A. Welch, H. A. Scheraga, Y. A. Arnautova, M. U. Schmidt, J. van de Streek, A. K. Wolf and B. Schweizer, *Acta Crystallographica Section B*, 2009, **65**, 107–125.
